# Investigating the mechanism of Xian-ling-lian-xia-fang for inhibiting vasculogenic mimicry in triple negative breast cancer via blocking VEGF/MMPs pathway

**DOI:** 10.1186/s13020-022-00597-5

**Published:** 2022-04-04

**Authors:** Feifei Li, Youyang Shi, Yang Zhang, Xiaojuan Yang, Yi Wang, Kexin Jiang, Ciyi Hua, Chunyu Wu, Chenping Sun, Yuenong Qin, Sheng Liu

**Affiliations:** Integrated Traditional Chinese and Western Medicine Breast Department, Longhua Hospital, Shanghai University of Traditional Chinese Medicine, Shanghai, 200030 China

**Keywords:** Chinese medicine decoction, Triple-negative breast cancer, Vascular mimicry, Network pharmacology, Experimental validation

## Abstract

**Background:**

Xian-ling-lian-xia-fang (XLLXF), a Chinese medicine decoction, is widely used in the treatment of triple negative breast cancer (TNBC). However, the underlying mechanism of XLLXF in TNBC treatment has not been totally elucidated.

**Methods:**

Here, network pharmacology and molecular docking were used to explore the mechanism of Traditional Chinese medicine in the treatment of TNBC. Then, biological experiments were integrated to verify the results of network pharmacology.

**Results:**

Network pharmacology showed that the candidate active ingredients mainly included quercetin, kaempferol, stigmasterol, and β-sitosterol through the “XLLXF–active ingredients–targets” network. Vascular endothelial growth factor A (VEGFA) and matrix metalloproteinase (MMP) 2 were the potential therapeutic targets obtained through the protein–protein interaction (PPI) network. Molecular docking confirmed that quercetin, kaempferol, stigmasterol, and β-sitosterol could stably combine with VEGFA and MMP2. Experimental verification showed that XLLXF could inhibit proliferation, colony ability, and vasculogenic mimicry (VM) formation and promote cell apoptosis in TNBC. Laser confocal microscopy found that XLLXF impaired F-actin cytoskeleton organization and inhibited epithelial mesenchymal transition. Animal experiments also found that XLLXF could inhibit tumor growth and VM formation in TNBC xenograft model. Western blot analysis and immunohistochemical staining showed that XLLXF inhibited the protein expression of VEGFA, MMP2, MMP9, Vimentin, VE-cadherin, and Twist1 and increased that of E-cadherin, tissue inhibitors of metalloproteinase (TIMP)-1, and TIMP-3 in vitro and in vivo.

**Conclusions:**

Integrating the analysis of network pharmacology and experimental validation revealed that XLLXF could inhibit VM formation *via* downregulating the VEGF/MMPs signaling pathway.

**Supplementary Information:**

The online version contains supplementary material available at 10.1186/s13020-022-00597-5.

## Introduction

Breast cancer is the most common malignant tumor in female. Latest statistics showed approximately 2.26 million new cases of breast cancer worldwide, accounting for roughly 11.7% of the world’s new cancer cases in 2020 [1]. Breast cancer is a highly heterogeneous tumor, in which TNBC is the most aggressive type. TNBC is prone to recurrence and metastasis because patients do not benefit from endocrine therapy and antihuman epidermal growth factor receptor-2 (HER-2) targeted therapy [2]. High invasiveness and limited therapeutic drugs of TNBC have led to a high 5-year mortality rate for patients with TNBC, which has become a difficult point in the treatment of breast cancer.

Angiogenesis is considered one of the poor prognostic factors affecting the survival of patients with TNBC. However, anti-angiogenic therapy by targeting key kinases in the angiogenesis process has not substantial improved the recurrence and prognosis of TNBC. The discovery of the VM phenomenon explained the failure of traditional anti-angiogenic therapies and resulted in the realization that tumors have another blood supply mode, which does not depend on vascular endothelial cells [3]. VM forms vascular-like structures through tumor cell self-deformation and extracellular matrix remodeling. The tumor cells covering the VM channel express some endothelial cell markers, suggesting that the VM process is similar to epithelial–mesenchymal transition (EMT) and involves transition from epithelial features to endothelial phenotypes. Studies have found that the VM phenomenon is more common in TNBC than in other types of breast cancer [4]. This finding may be related to its proneness to metastasis, recurrence, and adverse biological behavior of drug resistance.

Traditional Chinese medicine (TCM) could promote postoperative recovery, prevent breast cancer metastasis and recurrence, prolong survival time, and improve quality of life [5]. TCM believes that the pathogenesis of TNBC is based on “righteousness, deficiency, and evil.” When phlegm, blood stasis, and toxin are combined, they could disturb the wind of the liver. If the wind carries poisonous evils to other organs of the body, invasion and metastasis occur [6]. XLLXF is composed of *Codonopsis pilosula* Nannf., *Poria cocos* Wolf., *Epimedium brevicornu* Maxim., *Prunella vulgaris* L, *Curcuma phaeocaulis* Val., and *Scutellaria barbata* D. Don., formulated with the principles of “invigorating spleen and kidney, detoxification, and dispelling wind.” Previous clinical studies have proven that XLLXF could effectively prolong disease free survival and control the metastasis of patients with TNBC [7–9]. Pharmacological studies have confirmed the multitarget tumor suppressor effect of XLLXF to inhibit signal transduction and cancer cell invasion and metastasis [10–15].

The concept of network pharmacology was first proposed by the British scholar Hopkins in 2007 [16]. In recent years, the research on network pharmacology has become increasingly popular, providing new measures for the interpretation of the mechanism of TCM [17]. Research on breast cancer treatment by TCM based on network pharmacology has been realized through the exploration of “multitarget and multicomponent” at the molecular level [18, 19].

**Fig. 1:**
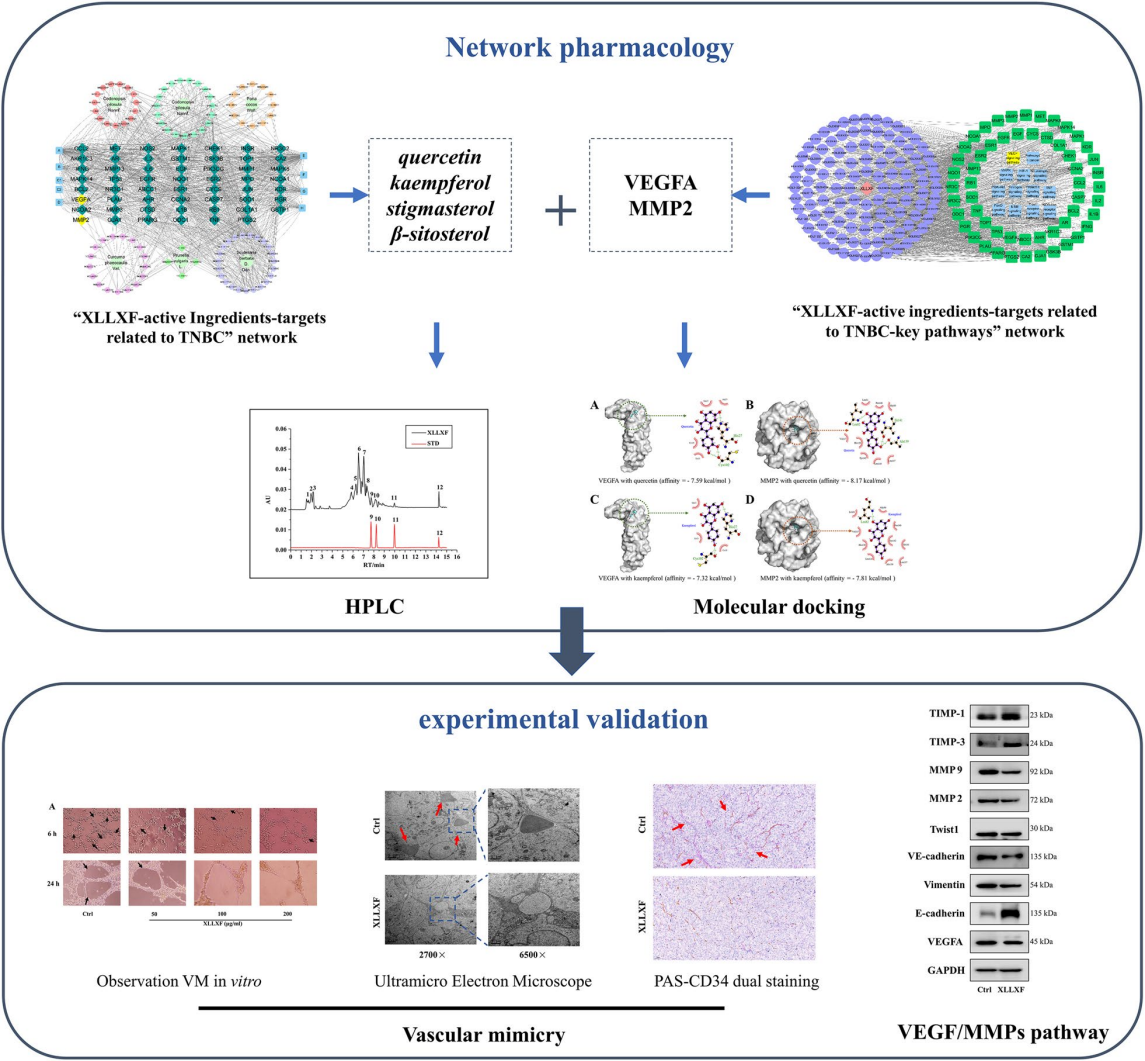
Flowchart of the molecular mechanism of XLLXF in treating TNBC

In this study, “XLLXF–active ingredients–targets” network was first constructed on the basis of active ingredients and common targets screened from databases. Then, candidate active ingredients were obtained by the degree of network, and potential therapeutic targets were obtained through the protein–protein interaction (PPI) network. Second, enrichment analysis was conducted on the intersection targets to screen the pathways of XLLXF in the treatment of TNBC. Third, molecular docking was performed to verify the active ingredients and target proteins, which play important roles in signaling pathways related to TNBC. Next, quercetin, kaempferol, stigmasterol, and β-sitosterol in XLLXF were unambiguously identified by comparing the retention time with that of standard samples by high-performance liquid chromatography (HPLC). Finally, a series of experiments was conducted to verify the previous hypothesis in vivo and in vitro. Data excavation and close experiments were performed to explore the specific molecular mechanism of XLLXF in the treatment of TNBC. A schematic of the work procedures is exhibited in Fig. 1.

## Methods

### Drug preparation

*Codonopsis pilosula* Nannf., *Poria cocos* Wolf., *Epimedium brevicornu* Maxim., *Prunella vulgaris* L, *Curcuma phaeocaulis* Val., and *Scutellaria barbata* D. Don. were provided by Shanghai Kang Qiao Chinese Cut Crude Drug Company and identified by WUXI APPTEC (SHANGHAI) Co., Ltd (China). Morphological, microscopic, and phytochemical identification were performed in accordance with the Pharmacopoeia of the People’s Republic of China (2015 edition). *Codonopsis pilosula* Nannf. (12 g), *Poria cocos* Wolf. (12 g), *Epimedium brevicornu* Maxim. (15 g), *Prunella vulgaris* L. (9 g), *Curcuma phaeocaulis* Val. (30 g), and *Scutellaria barbata* D. Don. (30 g) of XLLXF were weighed and added to a stainless pot. After 1080 mL of distilled water was added and boiled for 1 h, liquid was collected. Then, 1080 mL of water was added and boiled for another 1 h to collect liquid again. The solution was combined twice, a rotary evaporator was used to concentrate the solution, and the final volume was concentrated to 500 ml. The solution was combined and concentrated twice, and the concentrated extract was freeze dried to obtain the XLLXF extract at a yield of 29.23% (w/w, dried extract/crude herbs).

### Collection of active ingredients and targets of XLLXF

The active ingredients of *Codonopsis pilosula* Nannf., *Poria cocos* Wolf., *Epimedium brevicornu* Maxim., *Prunella vulgaris* L, *Curcuma phaeocaulis* Val., and *Scutellaria barbata* D. Don. in XLLXF were searched through the TCMSP database (https://tcmspw.com/tcmsp.php). *Curcuma phaeocaulis* Val. was used, with oral bioavailability (OB) ≥ 30% and drug likeness (DL) ≥ 0.10 as the screening conditions, while the others had screening conditions of OB ≥ 30% and DL ≥ 0.18. Relevant databases, such as CNKI and PubMed, were consulted to supplement the key herbal-related active ingredients screened in the TCMSP database. TCMSP was also used to obtain related targets, and then gene symbols were acquired from UniProt database (https://www.uniprot.org).

### Collection of targets between XLLXF and TNBC

With “triple negative breast cancer” as the search term, the TNBC-related targets were selected by the Gene Cards database (https://www.genecards.org). A Venn diagram was drawn to clarify the interaction between the TNBC-related targets and the potential targets of XLLXF.

### Construction of “XLLXF–active ingredients–targets” network and PPI network

The “XLLXF**–**active ingredients**–**targets” network was drawn by Cytoscape 7.2. Its built-in tool network analyzer was used to calculate the degree, and the main active ingredients were screened out. The PPI network was constructed using the string database (https://string-db.org). Modular clustering of the protein network was conducted to obtain core proteins with higher degrees by using the MCODE plug-ins in Cytoscape 7.2.

### Gene Ontology (GO) and Kyoto Encyclopedia of Genes and Genomes (KEGG) pathway enrichment analysis

GO and KEGG pathway enrichment analysis was performed through the DAVID database (https://david.ncifcrf.gov). On the basis of gene counts, the top 10 processes were correspondingly shown as bubble charts. The “XLLXF–active ingredients–targets–key signal pathway” network was drawn by Cytoscape 7.2.

### Molecular docking

The 3D crystal structures of the core targets were extracted from the Protein Data Bank (http://www.rcsb.org/pdb/). Then, the protein structures were processed by AutoDock Tools, including removal of ligands and water molecules, calculation of Gasteiger charge, addition of polar hydrogen, and combination of non-polar hydrogen. Subsequently, molecular docking was carried out *via* AutoDock Vina. Finally, the receptor–ligand complex was imported into Ligplus software to analyze the hydrogen bonding and hydrophobic interaction between the receptor and the ligand.

### Cell lines and cell proliferation assays

Human TNBC cell line MDA-MB-231 was purchased from the Chinese Academy of Sciences (Shanghai, China) and cultured in Dulbecco’s modified Eagle medium (DMEM) containing with 10% fetal bovine serum and 1% penicillin/streptomycin in a saturated humidity environment at 37 °C and 5% CO_2_

The proliferation of MDA-MB-231 cells was detected by 3-(4,5)- dimethylthiazolyl-3,5-diphenyltetrazolium bromide (MTT) assay. In brief, the cells were blown into a single-cell suspension (1.0 × 105 cells/mL) and seeded into 96-well plates (100 µL/well). Twenty-four h later, the culture medium was replaced with fresh medium containing various concentrations of XLLXF (0, 6.25, 12.5, 25, 50, 100, and 200 µg/mL). The cells were then cultured with a drug-containing medium for 24, 48, and 72 h. Afterwards, the MTT solution was added into the 96-well plates and incubated for 4 h at 37 °C. Optical density (OD) was detected at 490 nm with a BioTek instrument (Winooski, Vermont, USA). Data were expressed as the mean ± SD of at least three independent experiments.

### Colony formation assays

Colony formation assays were performed to further determine the inhibitory effect of XLLXF on the tumorigenicity of TNBC cells. A total of 500 MDA-MB-231 cells were seeded into six-well plates to incubate overnight. The cells were incubated with different final concentrations (0, 50, 100, and 200 µg/mL) of XLLXF for 7 days. After fixing with 4% paraformaldehyde and staining with a crystal violet solution, colonies containing more than 30 individual cells were counted under a stereomicroscope.

### Cell apoptosis assay

The apoptosis in MDA-MB-231 cells was measured by TUNEL and JC-1 with the use of a one-step TUNEL apoptosis assay kit (Beyotime, China) and a mitochondrial membrane potential assay kit with JC-1 (Beyotime, China). Log-phase MDA-MB-231 cells were seeded in a six-well plate with small glass slides in advance and randomly divided into control group and XLLXF groups (50, 100, and 200 µg/mL). After treatment for 24 h, TUNEL staining was performed in the TUNEL experiment. One mL of JC-1 staining working solution was added in the JC-1 experiment and incubated at 37 °C for 30 min in a cell incubator. Then, the cells were washed twice with JC-1 staining buffer and added with 2 mL of DMEM. Images were captured by a confocal laser microscope.

### Phalloidin dying of F-actin

Log-phase MDA-MB-231 cells were harvested and resuspended. A total of 200 µL of suspended cells was then pipetted into 35 mm chamber slides at a density of 1 × 10^5^/mL. After being subjected to treatments with DMEM or XLLXF (100 µg/mL) for 24 h, the cells were fixed with 4% paraformaldehyde in cytoskeleton for 30 min at room temperature. Then, they were permeabilized with 0.5% Triton X-100 for 5 min and blocked with BSA for 1 h at room temperature. Between each step described above, the cells were washed three times with PBS at 5 min each. F-actin was stained with Alexa Fluor 488 Phalloidin for 1 h to visualize the actin cytoskeleton. The cells were then counterstained with 4 mg/mL of 4,6-diamidino-2-phenylindole (DAPI).

### Immunofluorescence experiment

The treated MDA-MB-231 cells were fixed in 4% paraformaldehyde for 5 min, permeabilized with 0.5% Triton X-100 for 5 min, and blocked with BSA for 1 h at room temperature. Then, they were incubated with primary antibodies at 4 °C overnight, followed by incubation with fluorophore-conjugated secondary antibody for 1 h. The samples were stained with DAPI and imaged using a confocal microscope after washing three times.

### Three-dimensional cultures

A 24-well tissue culture plate was evenly coated with 200 µL/well growth factor-reduced Matrigel, which was allowed to solidify at 37 ℃ for 60 min before cells were plated. The cell suspension was added (1 × 105 cells/well) onto the surface of the Matrigel and incubated at 37 ℃ for 24 h. Net-like structures lined with tumor cells were considered the mimicking vessels.

### Western blot analysis

After the MDA-MB-231 cells were treated with XLLXF (50, 100, and 200 µg/mL) for 24 and 48 h, total cell protein lysates were extracted using RIPA lysis buffer that contained protease and phosphatase inhibitor cocktails. Protein lysates (20 µg), which were determined by BCA analysis (Beyotime, China), were loaded onto 10% SDS-PAGE gels. The protein bands were transferred onto NC membranes and blocked with 5% non-fat milk for 1 h at room temperature. The NC membranes with proteins were incubated with diluted primary antibodies at 4 °C overnight. The primary antibodies used in the analyses were as follows: VEGFA (1:1,000, Proteintech), MMP2 (1:1000, Proteintech), MMP9 (1:1000, Cell Signaling Technology), Vimentin (1:1000, Proteintech), VE-cadherin (1:1,000, Cell Signaling Technology), TIMP-1 (1:1000, Proteintech), TIMP-3 (1:1,000, Proteintech), and Twist1 (1:1000, Proteintech). The membranes were incubated with relative sources of secondary antibodies (1:5000) at room temperature for 1 h. Specific protein bands were recognized with Immobilon Western Chemiluminescent HRP Substrate (Millipore, MA, USA). Image J software was used for image analysis.

### Tumor xenograft assay in vivo

Seven-week-old female nude BALB/c mice (18–20 g) were obtained from Shanghai SLAC Laboratory Animal Technology Co., Ltd (Shanghai, China). The protocol was approved by the Animal Research Ethics Committee of Shanghai University of Traditional Chinese Medicine. MDA-MD-231 cells (1 × 10^7^) premixed with Matrigel at a ratio of 1:1 was subcutaneously injected into the fourth pair of breast fat pads on the left side of each mouse. The tumors formed approximately 7 days after the inoculation. XLLXF (18 g/kg) was administered intragastrically; it was converted in accordance with the 10-fold effective dose between human (60 kg) and mice (20 g). All mice were then randomly divided into two groups (n = 5): control group (i.g., saline) and XLLXF group (i.g., 18 g/kg). The mice received corresponding treatment once a day for 4 weeks. Tumor volumes were calculated using the following formula: V = 0.5 × a × b^2^, where V denotes tumor volume, a denotes maximum tumor diameter, and b denotes minimum tumor diameter. Body weight was measured once a week as an indicator to assess the animals’ overall health. The mice were euthanized upon the experiments’ termination.

### Electron microscopy

Xenograft tissues (0.5 mm^3^) were fixed in cold 2.5% glutaraldehyde in 0.1 M sodium cacodylate buffer and postfixed in 1% osmium tetroxide, dehydrated, and embedded in a standard fashion for transmission electron microscopy. The specimens were then embedded, sectioned, stained, and observed using Hitachi HT-7700 TEM.

### Immunohistochemical (IHC) staining

The slides were deparaffinized twice with xylene for 10 min and rehydrated with 100–75% ethanol for 10 min. After the slices were washed with PBS three times, they were boiled in 10 mm sodium citrate buffer solution for 8 min for antigen repair. Sections were permeabilized with 3% hydrogen peroxide dissolved in methanol at room temperature in the dark to eliminate endogenous peroxidase activity and then blocked by 10% goat serum to reduce nonspecific binding. The samples were then washed with PBS three times and incubated with 1:200 diluted primary antibodies in a humid chamber at 4 °C overnight, followed by incubation with a 1:200 dilution of biotinylated secondary antibodies. Immediately thereafter, 3,3-diaminobenzidine substrate was applied for color development, and counterstaining with Mayer’s hematoxylin was performed.

### PAS-CD34 dual staining

The procedure was the same as the above description in the IHC part. In brief, after DAB reaction, sections were treated with 0.5% periodic acid solution for 10 min and rinsed with distilled water for 5 min, followed by staining in Schiff solution for 15–30 min. After the sections were rinsed with distilled water, they were counterstained with hematoxylin, dehydrated, cleared, and mounted.

### ELISA

The levels of VEGFA and MMP2 were determined by ELISA kits (mlbio, shanghai, China). The serum of mice was specifically collected; 20 µL of serum, 30 µL of sample dilution, and 100 µL of HRP were added to each well of the antibody-coated plate for 1 h at 37 °C. Then, the liquid was removed, and the microplate was washed three times. Afterwards, 50 µL of the reaction solution was added to each well for 30 min at 37 °C with light prevention. Next, 50 µL of stop solution was added, and the absorbance was measured at 450 nm.

### Statistical analyses

SPSS software (version 25.0) was used for statistical analysis, and GraphPad Prism software (version 8) was used for plotting. All images were measured by ImageJ software. Measurement data conformed to a normal distribution, and they were compared *via* independent sample t-test analysis for two groups. One-way ANOVA was used for multigroup analysis. Nonparametric rank sum test was used if the measurement data did not conform to a normal distribution. P < 0.05 was considered statistically significant.

## Results

### Active ingredients of XLLXF and their putative targets related to TNBC

A total of 110 chemical compounds from six drugs constituting XLLXF were screened in the TCMSP databases, including 17 from *Codonopsis pilosula* Nannf., 14 from *Poria cocos* Wolf., 19 from *Epimedium brevicornu* Maxim., 23 from *Curcuma phaeocaulis* Val., 24 from *Scutellaria barbata* D. Don., four from *Prunella vulgaris* L, and nine common chemical compounds (Additional file 1: Table S1).

**Fig. 2:**
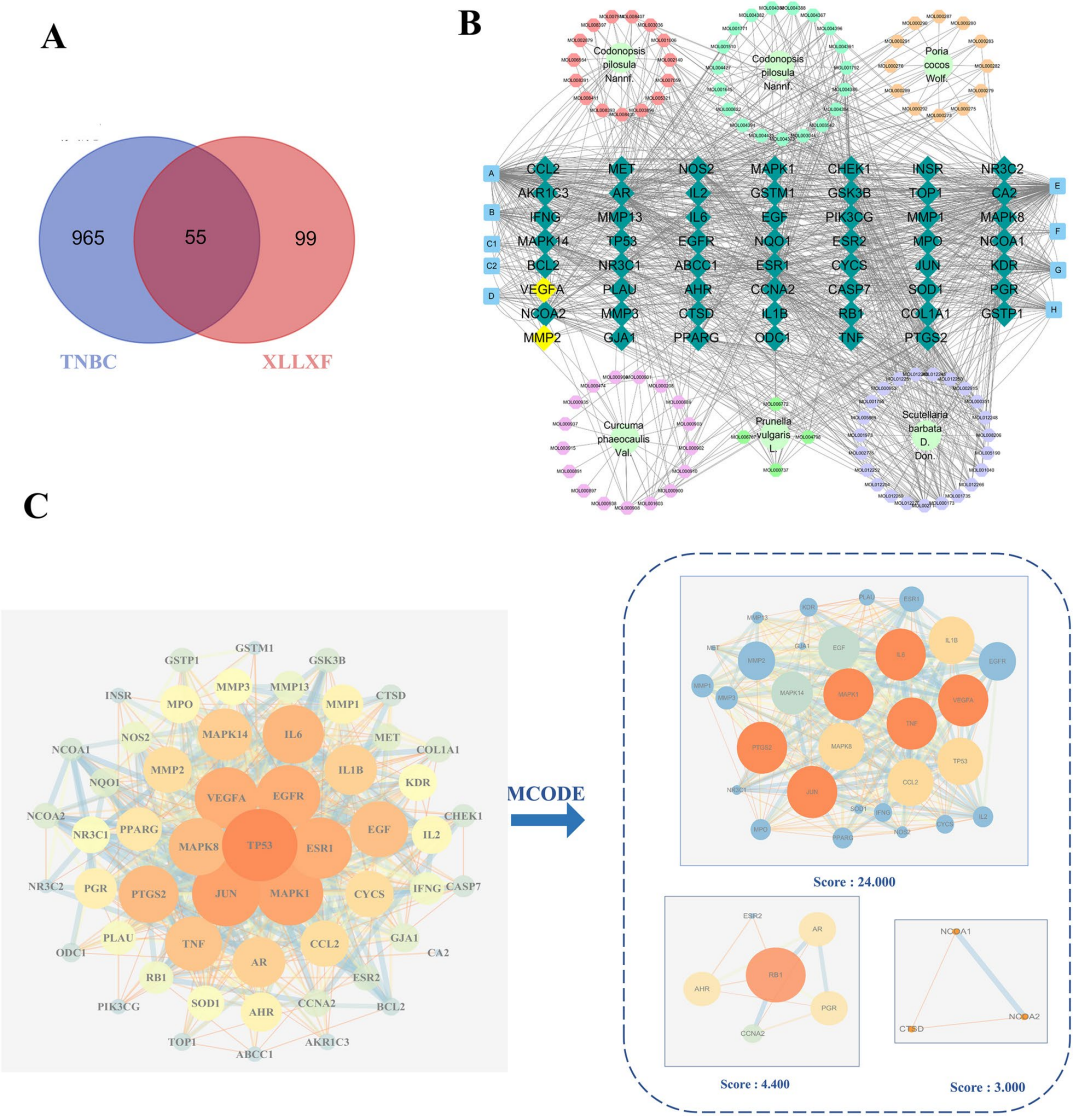
“XLLXF–active ingredients–targets related to TNBC” network and PPI network analysis of common targets. **a** Interactive targets between XLLXF and TNBC. **b** “XLLXF–active ingredients–targets related to TNBC” network. Yellow rectangles represent VEGFA and MMP2. **c** PPI network of common targets and MCODE analysis

Through the TCMSP and Uniprot database, a total of 154 target genes of active ingredients were obtained. Then, 1020 TNBC-related targets were screened through the GeneCards database. In addition, 55 interactive targets between XLLXF and TNBC were identified (Additional file 2: Tables S2; Fig. 2a). The “XLLXF–active ingredients–targets related to TNBC” network was drawn by Cytoscape 7.2 (Fig. 2b). Network Analyzer calculated 160 nodes and 1091 edges, with an average degree value of 10. The top active ingredients were screened in accordance with the degree value. Among them, the compounds quercetin, kaempferol, stigmasterol, and β-sitosterol were the candidate active ingredients in XLLXF (Additional file 3: Table S3).

### PPI network

PPI networks were constructed through the String database. For intuitive topological analysis, 55 nodes and 691 edges were obtained, with average and median degrees of 22.51 and 21. The degree values of VEGFA and MMP2 ranked higher than those of others; thus, they may be potential therapeutic targets. MCODE analysis showed three main protein clustering modules. Module one mainly involves RNA polymerase II promoter transcription and DNA binding transcription factor activity, regulation, cell proliferation, cell apoptosis, angiogenesis, and other processes. Module two involves intracellular receptor signaling pathways, androgen receptor signaling pathways, the negative regulation of epithelial cell proliferation, Ras protein signal transduction, the negative regulation of gene expression, viral process, cell division, and other processes. Module three involves the cell lipid metabolism process, the cell response of bile acid and bile salt transport, the intracellular receptor signal transduction pathway, and the cell response process to hormone stimulation (Fig. 2c).

### GO and KEGG pathway enrichment analysis

**Fig. 3:**
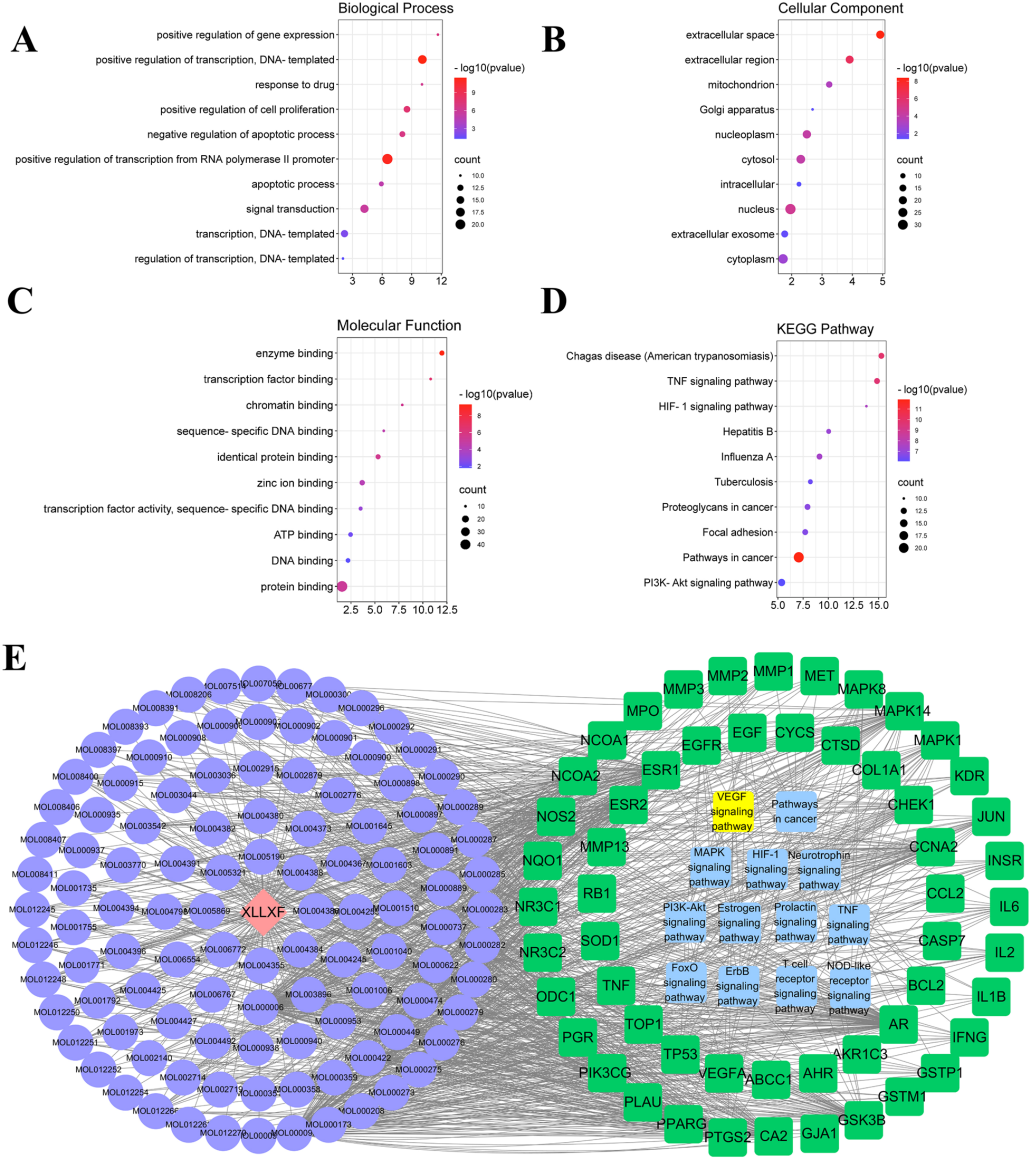
GO and KEGG enrichment analysis of common targets. **a** Bubble chart of the top 10 biological processes according to gene counts. **b** Bubble chart of the top 10 cellular component processes according to gene counts. **c** Bubble chart of the top 10 biological processes according to gene counts. **d** Bubble chart of the top 10 signaling pathway according to gene counts. **e** “XLLXF–active ingredients–targets related to TNBC–key pathways” network. Yellow rectangle represents VEGF signaling pathway

GO and KEGG pathway enrichment analyses were performed through the DAVID database. A total of 270 biological process entries were obtained, including RNA polymerase II promoter transcription, DNA transcription, signal transduction, cell proliferation, apoptosis, gene expression, drug response, and angiogenesis (Fig. 3a). A total of 28 cellular component entries were also obtained, including nucleus, cytoplasm, extracellular space, exosomes, mitochondria, Golgi apparatus, extracellular matrix, protein complexes, and lysosomes (Fig. 3b). In addition, 56 molecular function items were related to protein binding, zinc ion binding, enzyme binding, the same protein binding, ATP binding, sequence-specific DNA binding, transcription factor binding, chromatin binding, cytokine activity, and other functions (Fig. 3c). A total of 88 pathways were obtained by KEGG pathway analysis. The results indicated that XLLXF may play an anti-TNBC role, mainly through cancer-related pathways, PI3K/Akt signaling pathways, TNF signaling pathways, HIF-1 signaling pathways, MAPK signaling pathways, and VEGF signaling pathways (Fig. 3d). The “XLLXF–active ingredients–targets related to TNBC–key pathway” network was constructed by Cytoscape 7.2 (Fig. 3e).

### HPLC profile of XLLXF

**Fig. 4:**
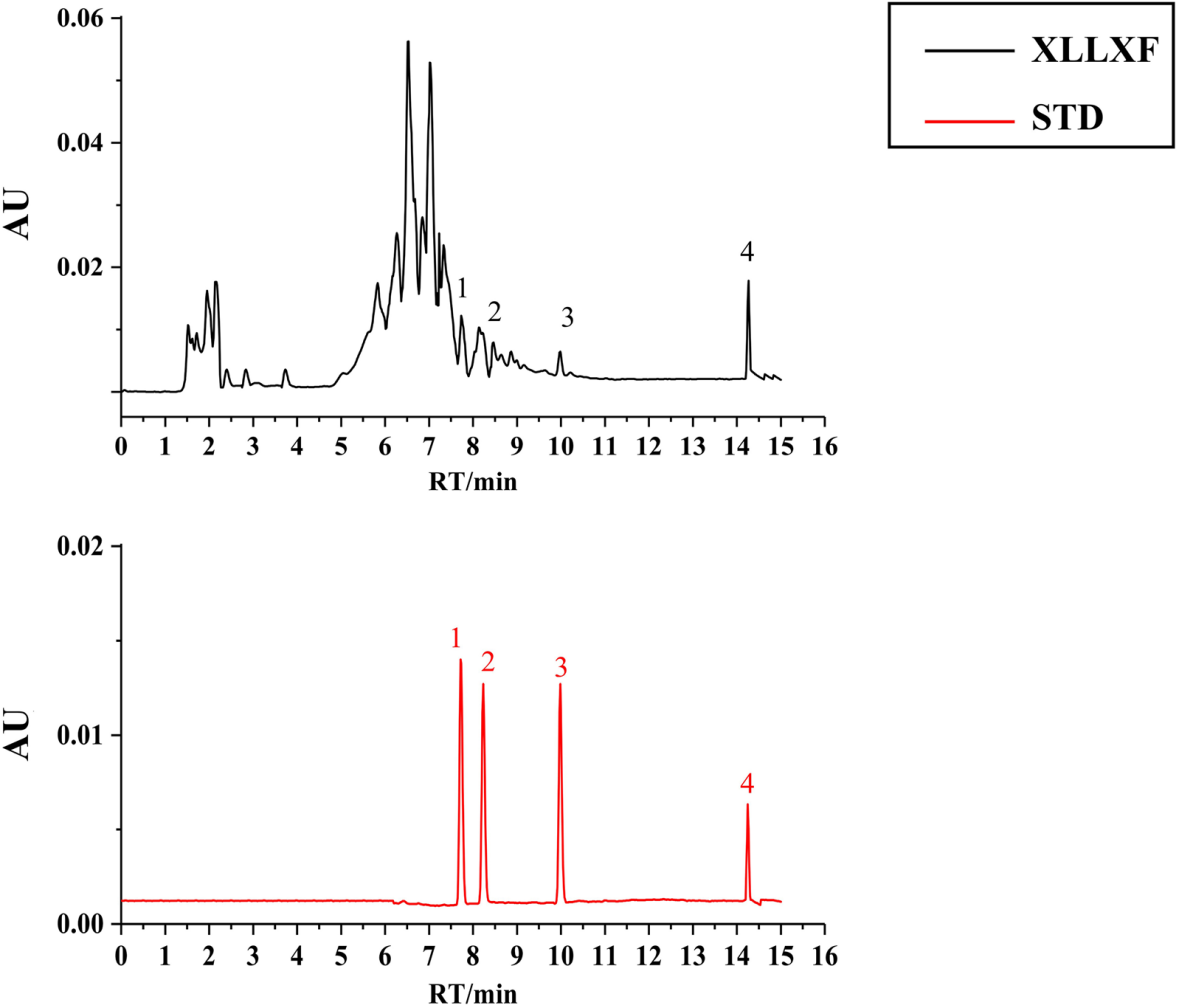
HPLC of XLLXF and standard substances. Black and red represent XLLXF and standard substances, respectively. Four identified compounds, including *quercetin* (peak 1), *kaempferol* (peak 2), *stigmasterol* (peak 3), and *β-sitosterol* (peak 4)

On the basis of the results of network pharmacology, quercetin, kaempferol, stigmasterol, and β-sitosterol were used as marker components for the quality control of XLLXF. They all were unambiguously identified by comparing the retention time with that of standard samples (Fig. 4). Detailed information and structures of these components are provided in Additional file 4: Table S4.

### Molecular docking verification

**Fig. 5:**
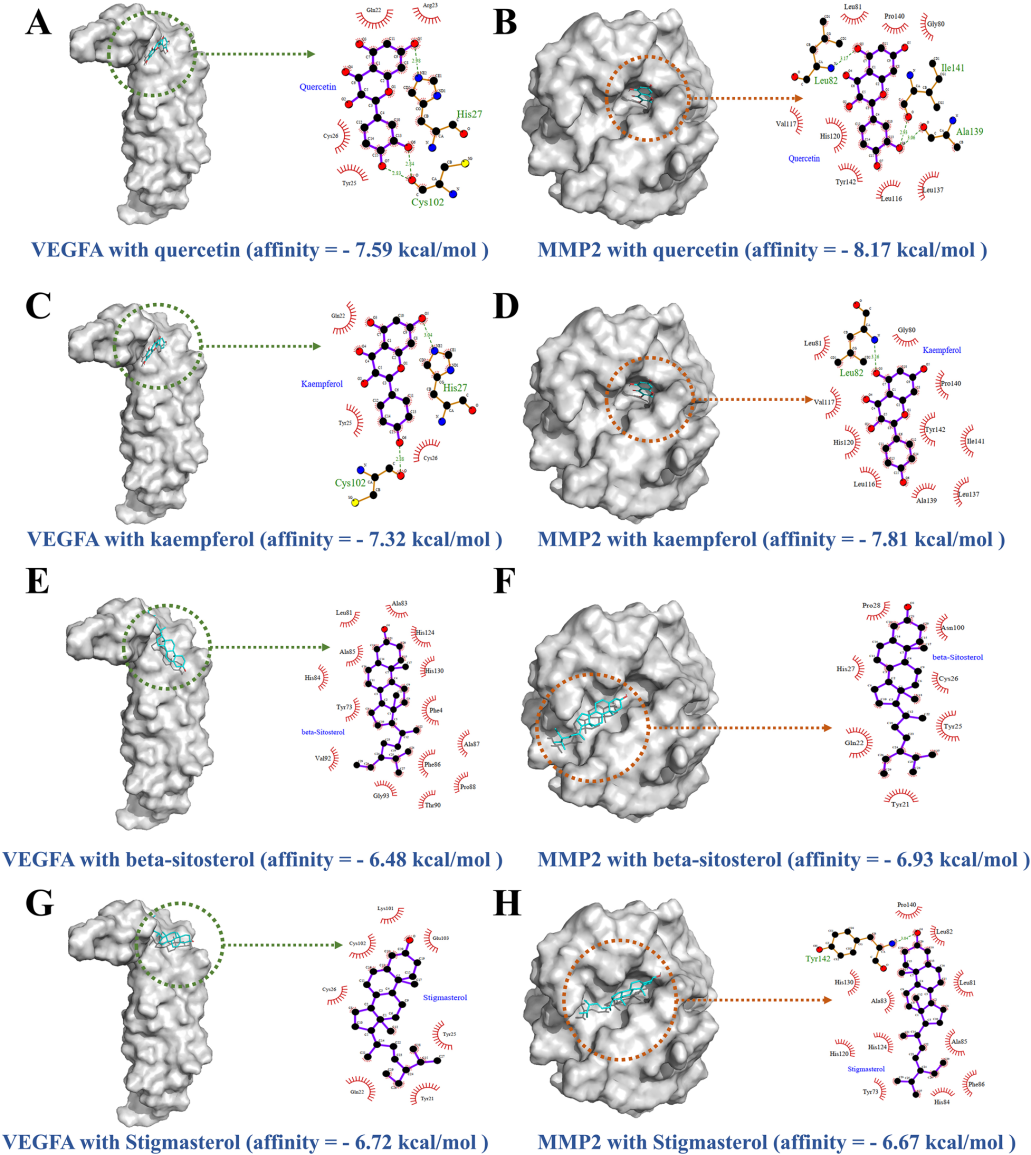
Molecular docking of core targets with corresponding compound. **a** *Quercetin* and VEGFA; **b** *quercetin* and MMP2; **c** *kaempferol* and VEGFA; **d** *kaempferol* and MMP2; **e** *β-sitosterol* and VEGFA; **f** *β-sitosterol* and MMP2; **g** *stigmasterol* and VEGFA; and **h** *stigmasterol* and MMP2

The candidate active ingredients quercetin, kaempferol, stigmasterol, and β-sitosterol and the potential therapeutic targets VEGFA and MMP2 were obtained by network pharmacology. Docking verification of the compounds and target proteins was performed through molecular docking technology. The results showed that the compounds and target proteins had a strong binding effect, and the binding energies were all less than − 6 kcal/mol (Additional file 5: Table S5). The most stable connective patterns and details of the binding energies are listed in Fig. 5a–h. According to network analysis and molecular docking, the candidate targets for the experiment were VEGFA and MMP2, which are closely related to the invasion, metastasis, and formation of vascular mimicry in TNBC (Additional file 6).

### XLLXF inhibited the survival of MDA-MB-231 cells

**Fig. 6:**
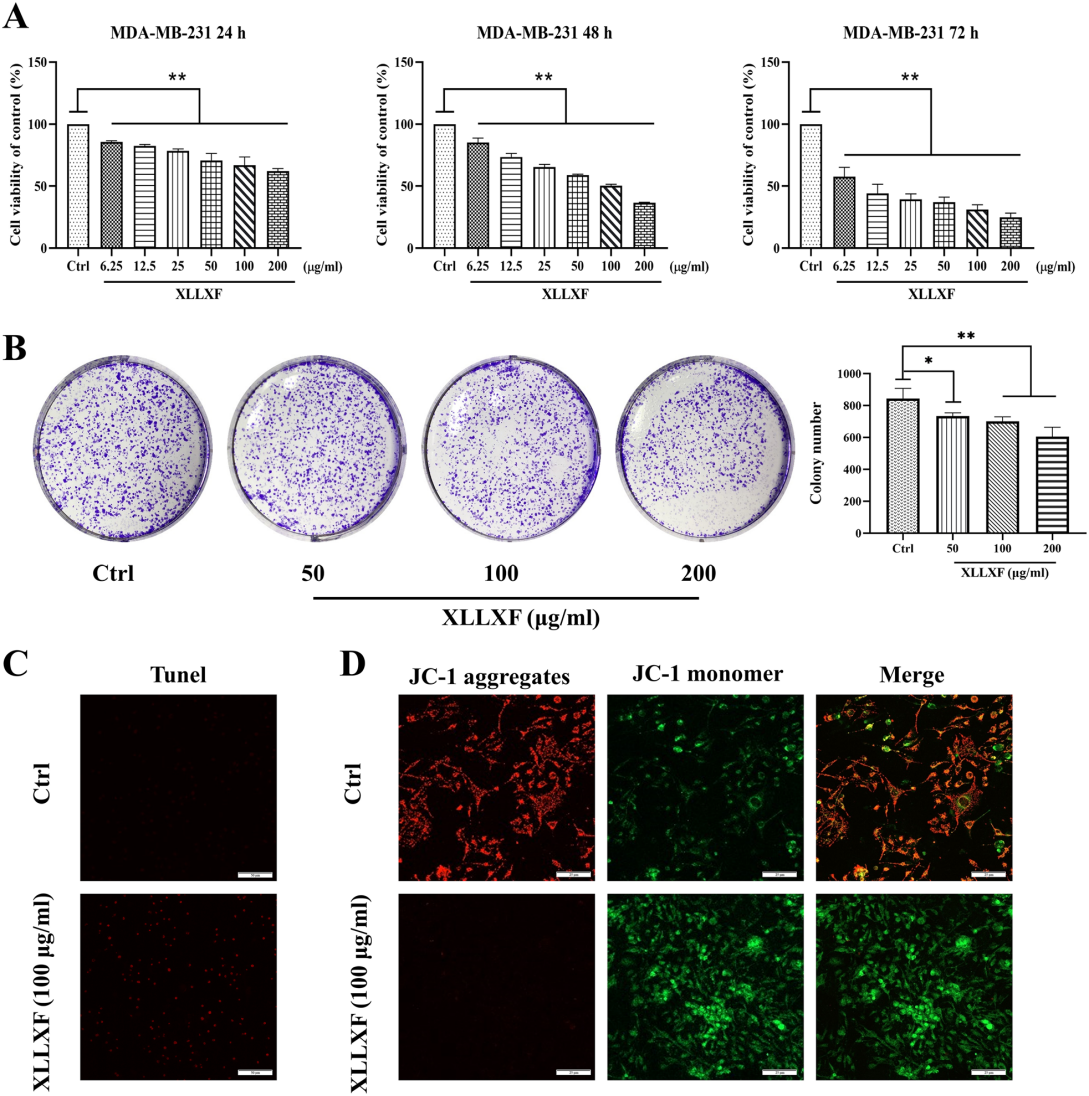
Inhibited survival of MDA-MB-231 cells by XLLXF. **a** Dose-inhibition curves of XLLXF at 24, 48, and 72 h. **b** XLLXF causing a reduction in colony number with the increase in concentration. **c** Immunofluorescent staining of TUNEL (200×) and apoptotic cells stained by red color. **d** Immunofluorescent staining of JC-1 (400×). Green fluorescence intensity gradually increased and red fluorescence intensity gradually decreased in the apoptotic cells

MTT assays showed that XLLXF inhibited the proliferation of MDA-MB-231 cells in a dose-and time-dependent manner (Fig. 6a). The IC50 values of XLLXF were 231.780, 123.532, and 9.807 µg/mL at 24, 48, and 72 h, respectively. This result was further confirmed by colony formation assay. The MDA-MB-231 cells treated by XLLXF showed a reduction in colony number with the increase in concentration compared with the control group, indicating that colony ability was inhibited (Fig. 6b).

The decrease in mitochondrial membrane potential is an important feature of the mitochondrial apoptotic pathway. When the mitochondrial membrane potential is high, JC-1 gathers in the matrix of the mitochondria to form aggregates and produce red fluorescence. When the mitochondrial membrane potential is low, JC-1 could not accumulate in the matrix of the mitochondria. At this time, JC-1 becomes a monomer and produces green fluorescence. Compared with the control group, after cells were treated with different concentrations (50, 100, and 200 µg/mL) of XLLXF for 24 h, the green fluorescence intensity gradually increased and the red fluorescence intensity gradually decreased with the increase in concentrations, suggesting that the mitochondrial membrane potential gradually decreased. Combination of the results of TUNEL staining and JC-1 assays revealed that XLLXF promoted the mitochondrial apoptotic pathway in MDA-MB-231 cells (Fig. 6c, d).

### XLLXF impaired F-actin cytoskeleton organization in MDA-MB-231 cells

**Fig. 7:**
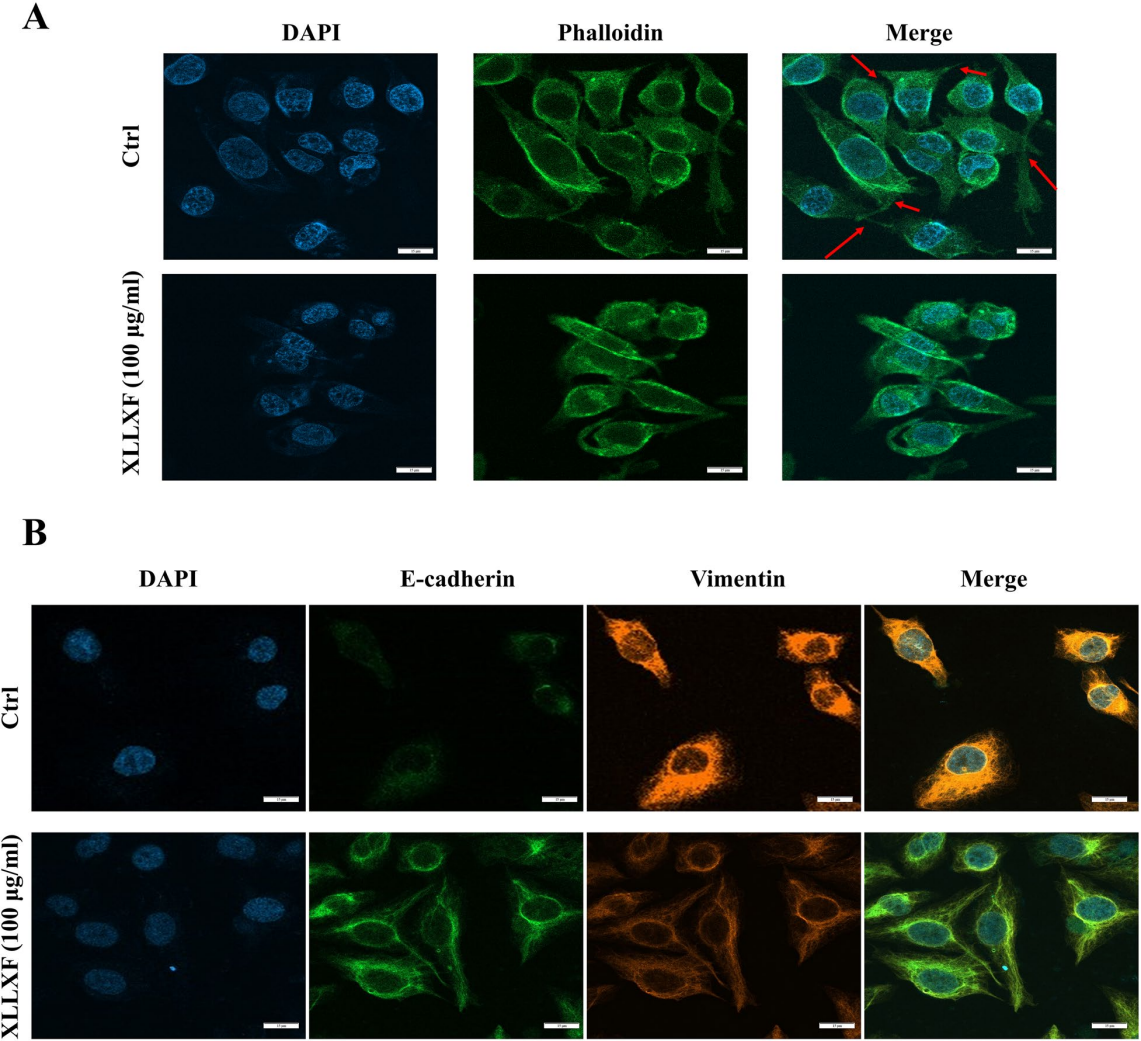
Impairment of F-actin cytoskeleton organization and inhibition of EMT in MDA-MB-231 cells by XLLXF. **a** XLLXF impaired F-actin fiber and the formation of lamellipodia in cells (red arrow). **b** XLLXF increased the expression of E-cadherin (green) and decreased that of Vimentin (orange) in MDA-MB-231 cells

The effects of XLLXF on F-actin microfilaments in MDA-MB-231 cells were investigated. The control cells exhibited a regular aggregation of F-actin present along the cells. When the cells were treated with 100 µg/mL XLLXF, a significant reduction in F-actin fiber expression and formation of lamellipodia in cells were observed (Fig. 7a).

### XLLXF increased the expression of E-cadherin and decreased the expression of Vimentin in MDA-MB-231 cells

Immunofluorescence experiment indicated that the E-cadherin protein expression in the XLLXF group was significantly upregulated, whereas Vimentin expression was significantly downregulated compared with those in the control group. This result indicated that XLLXF may significantly inhibit the occurrence and development of EMT (Fig. 7b).

### XLLXF inhibited VM formation in MDA-MB-231 cells ***via*** downregulating VEGF/MMPs signaling pathway in vitro

**Fig. 8:**
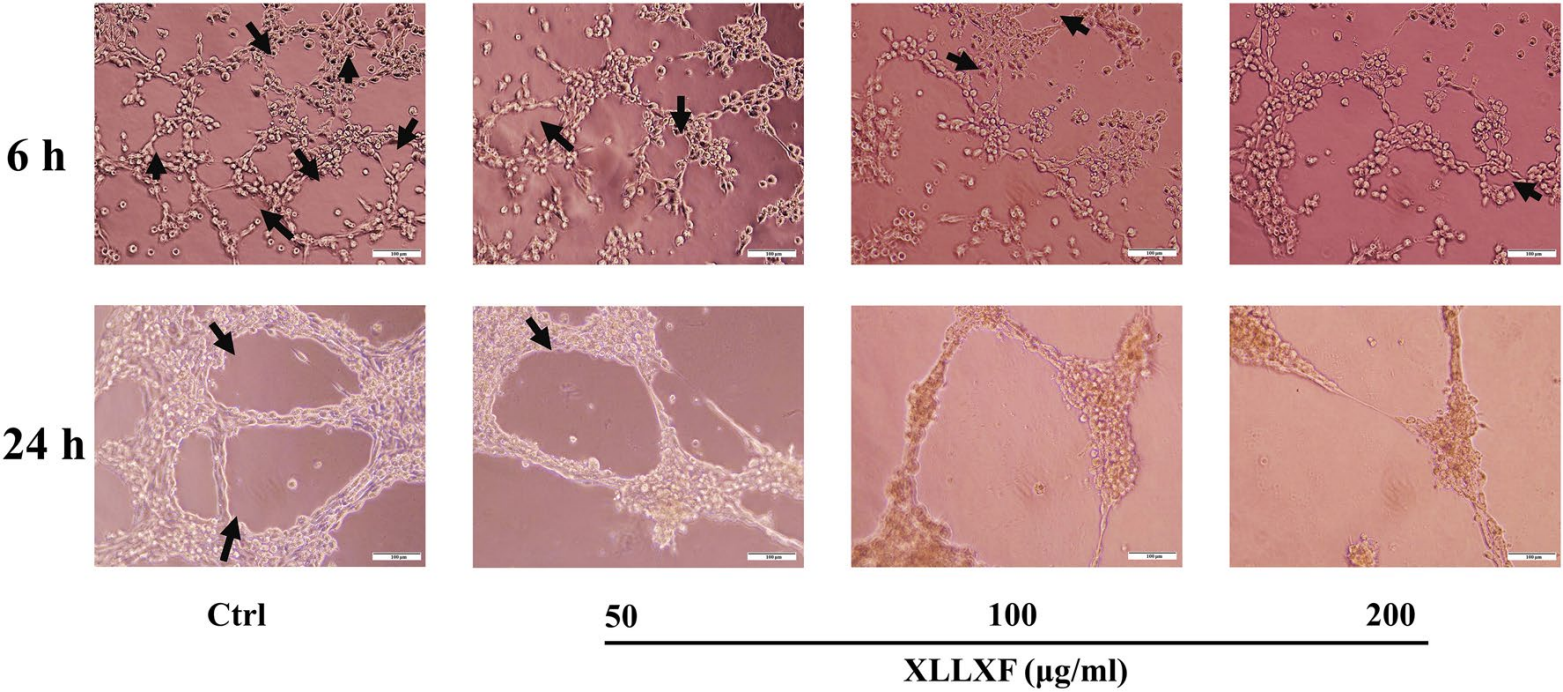
XLLXF inhibition of VM formation in MDA-MB-231 cells. Cells elongated and protruded pseudopodia to form net-like structures (black arrows), which were blocked by XLLXF treatment (400×)

Matrigel was used to establish the 3D culture system. On the Matrigel, MDA-MB-231 cells were proliferated in clusters. Cellular protrusions were obvious, and the cells formed connective structures resembling networks. After exposure to XLLXF, the intercellular connections were damaged, and net-like structures were reduced or they even disappeared in the high-concentration groups (100 and 200 µg/mL, Fig. 8).

**Fig. 9:**
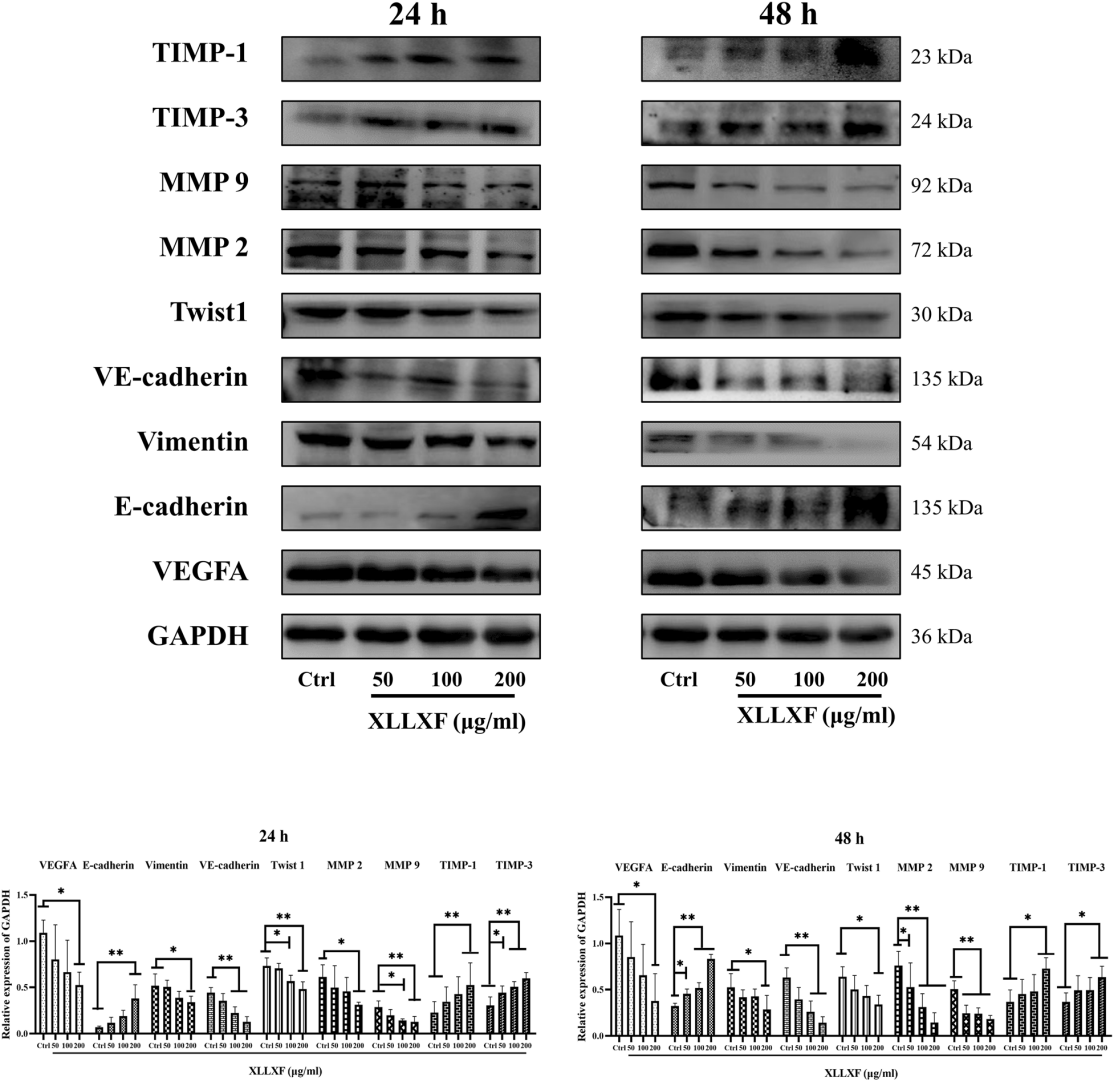
XLLXF inhibition of VEGF/MMPs signaling pathway in vitro. Western blot performed to compare the protein expression levels of VEGFA, MMP2, MMP9, Twist1, Vimentin, E-cadherin, VE-cadherin, TIMP1, and TIMP3 in MDA-MB-231 cells

In accordance with the results of network pharmacology, the expression levels of VEGFA, MMP2, MMP9, Twist1, Vimentin, E-cadherin, VE-cadherin, TIMP-1, and TIMP-3 in MDA-MB-231 cells treated with XLLXF were examined by Western blot. After being treated with 50, 100, and 200 µg/mL of XLLXF in 24 and 48 h, the expression levels of VEGFA, MMP2, MMP9, Twist1, Vimentin, and VE-cadherin were inhibited, whereas those of E-cadherin, TIMP-1, and TIMP-3 increased significantly in a dose- and time-dependent manner (Fig. 9). Collectively, the results suggested that XLLXF could affect VM formation in MDA-MB-231 cells via downregulating the VEGF/MMPs signaling pathway, which consistent with the results of network pharmacology.

### XLLXF inhibited tumor growth and VM formation in TNBC xenograft model

**Fig. 10:**
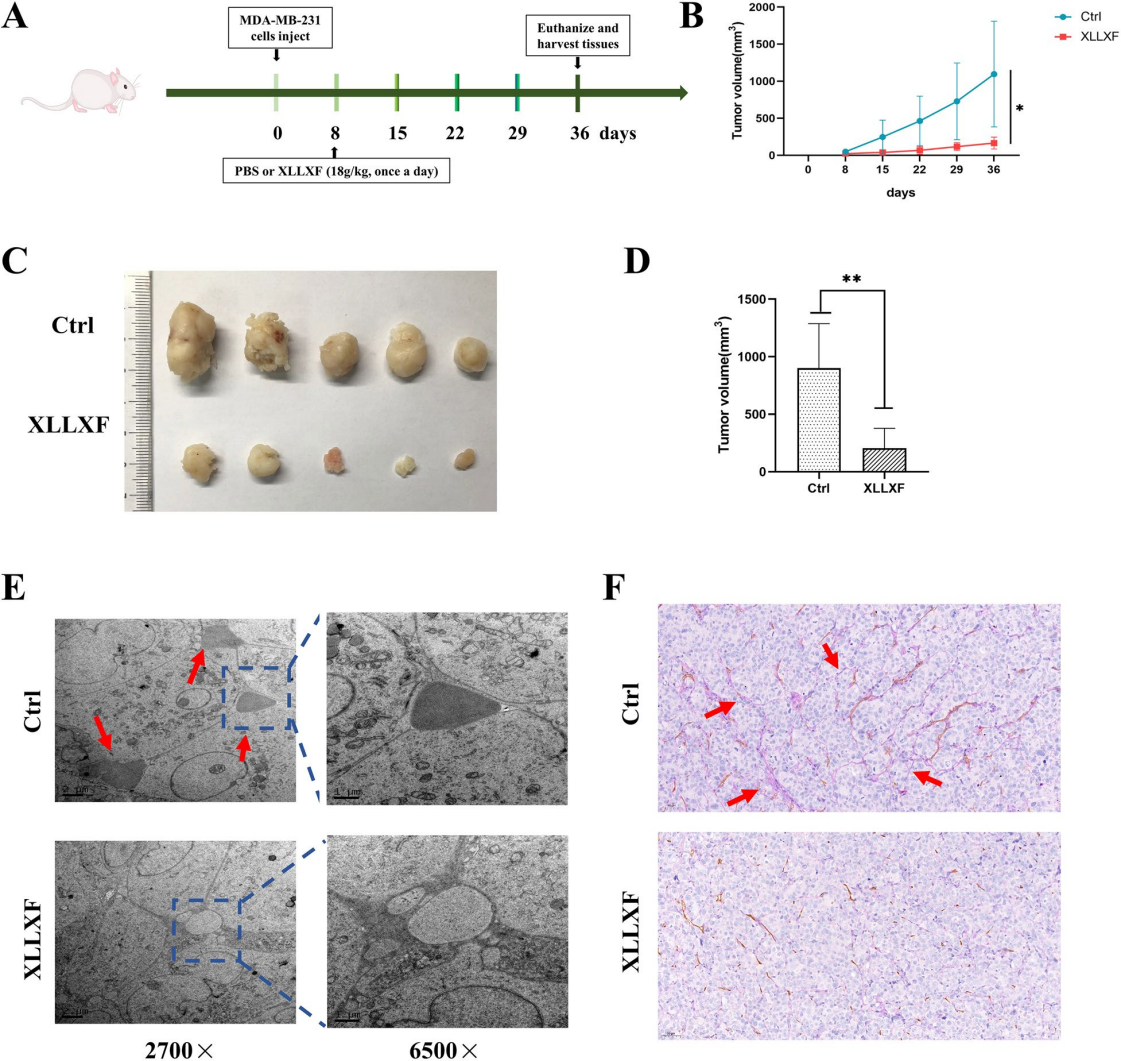
XLLXF inhibition of tumor growth and VM formation in TNBC xenograft model. **a** Schematic of tumor implantation and XLLXF treatment. **b** Tumor size was measured every 7 days from day 8 to day 36 after implanting MDA-MB-231 cells and calculated by length × width × width/2. **c**, **d** Picture of the tumor after mice were killed. The size of the tumor was also measured. **e** Ultrastructure of VM in each group, as shown by TEM. Red arrows indicate VM structures. Original magnifications were 2700× (left) and 6500× (right). **f** VM detection through CD34-PAS dual staining. Red arrows indicate VM structure with CD34-negative and PAS-positive network-like or vascular lumen structures. Original magnifications were 200×

On the basis of the above results in vitro, the effect of XLLXF in tumor growth and VM formation was examined in vivo. In the TNBC xenograft model, XLLXF was administered intragastrically at a dose of 18 g/kg starting from day 8 after tumor implantation until day 36 (Fig. 10a). The mice were sacrificed on day 36, and the tumor size was measured. In the TNBC xenograft model, XLLXF significantly delayed the tumor growth and reduced the tumor size compared with that in the control group (Fig. 10b–d).

Under the electron microscope, typical VM lined with red blood cells was found in the control group. In the XLLXF group, only vacuoles between the cells were observed, and no VM structure was found (Fig. 10e). VM structures are composed of tumor cells and extracellular matrix components. Thus, they lack the participation of vascular endothelial cells. The endothelial cells could be identified through specific surface molecule CD34 staining. The glycogen and collagen in the extracellular matrix could be stained into purple red by PAS staining. Therefore, CD34-negative and PAS-positive network-like or vascular lumen structures could be regarded as VM structures. In this study, CD34-PAS dual staining showed VM structures with negative CD34 staining and positive PAS staining in the tumors of the control group. Damaged VM structures and decreased VM quantity were observed in the XLLXF-treated groups (Fig. 10f).

### XLLXF inhibited VM formation ***via*** downregulating VEGF/MMPs signaling pathway in vivo

**Fig. 11:**
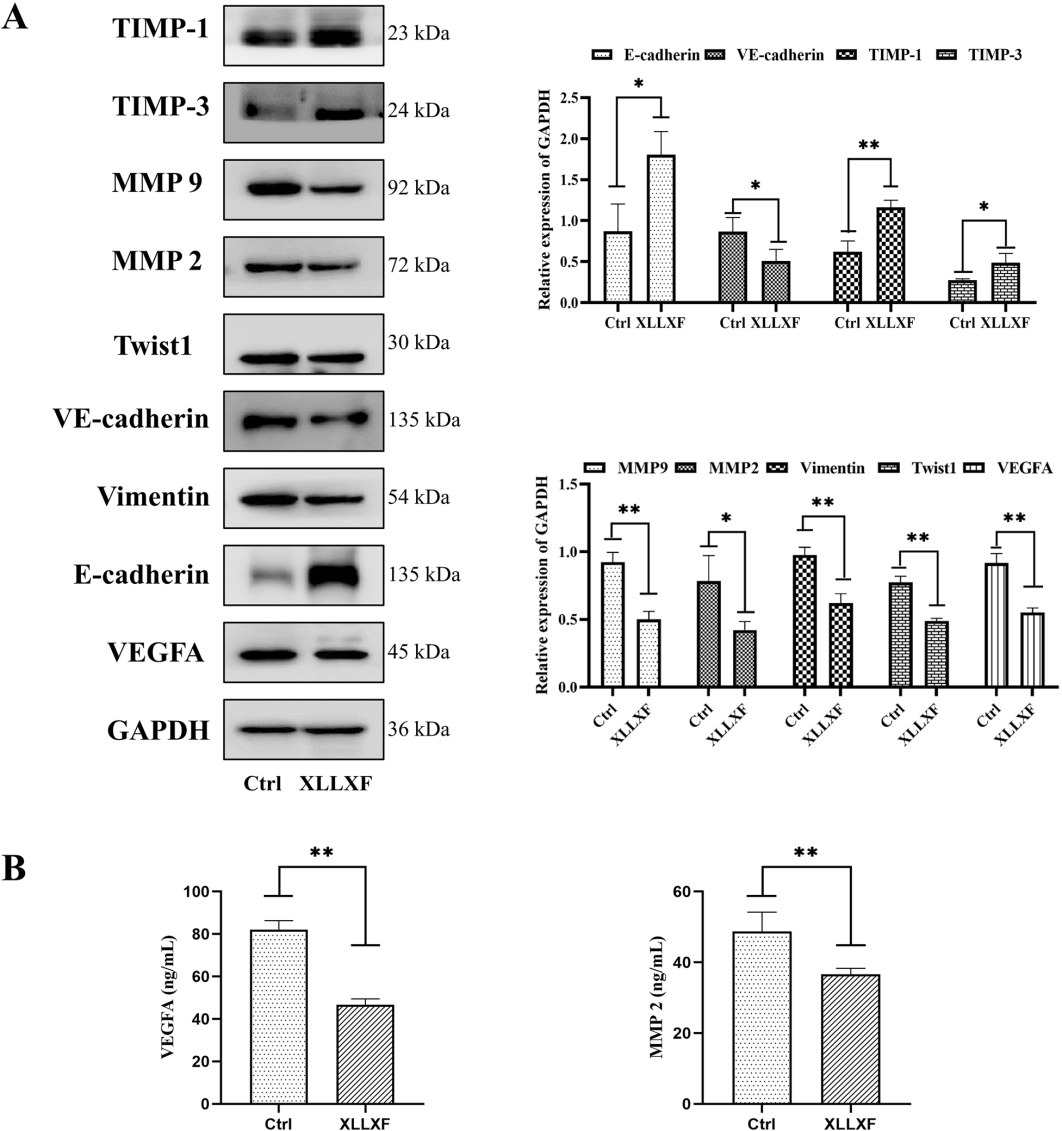
XLLXF inhibition of VEGF/MMPs signaling pathway in vivo. **a** Western blot performed to compare the protein expression levels of VEGFA, MMP2, MMP9, Twist1, Vimentin, E-cadherin, VE-cadherin, TIMP1, and TIMP3 in tumor tissues. **b** ELISA indicated the inhibitory effect of XLLXF on VEGFA and MMP2 in mouse serum

**Fig. 12:**
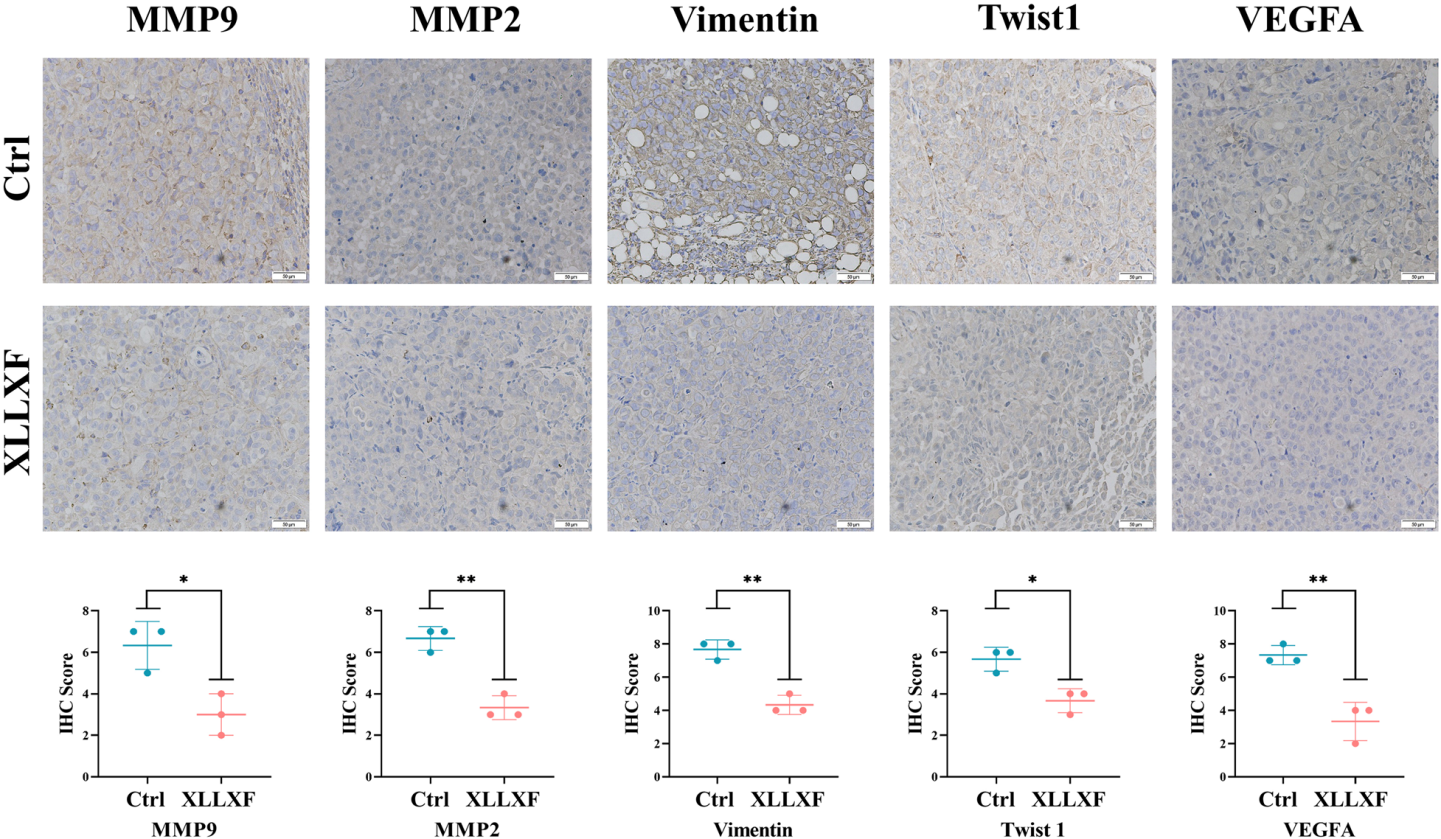
IHC staining performed to compare the expression of VEGFA, MMP2, MMP9, Twist1, and Vimentin. Original magnifications were 200×

Western blot was performed to compare the expression of VEGFA, MMP2, MMP9, Twist1, Vimentin, E-cadherin, VE-cadherin, TIMP-1, and TIMP-3 between the control group and the XLLXF group to further understand the molecular mechanism in *vivo*. The expression levels of VEGFA, MMP2, MMP9, Twist1, Vimentin, and VE-cadherin were inhibited significantly, whereas those of E-cadherin, TIMP-1, and TIMP-3 increased significantly in the XLLXF group (Fig. 11a). ELISA experiments also indicated the inhibitory effect of XLLXF on VEGFA and MMP2 (Fig. 11b). IHC staining showed a decrease in VEGFA, MMP2, MMP9, Twist1, and Vimentin in the XLLXF-treated groups (Fig. 12). These results indicated that XLLXF is able to inhibit VM formation *via* downregulating the VEGF/MMPs signaling pathway in vivo, consistent with the results of *in*-*vitro* experiments.

## Discussion

TNBC accounts for 10–15% of newly diagnosed breast cancers, in which the 5-year overall survival rate is only 76.5% [20]. Chemotherapy is currently the first choice of treatment for TNBC [21]. At present, chemotherapeutic treatment based on taxanes and anthracyclines is the standard regimen for early TNBC [22, 23], including the use of taxanes and anthracyclines in the intensive dosing regimen [24] or the addition of capecitabine [25, 26] or platinum [27, 28]. In addition, some new treatments have emerged, such as immunotherapy [29, 30], polyadenylic acid diphosphate ribose polymerase inhibitors [31], anti-angiogenesis drugs [32], and PI3K/Akt/mTOR inhibitors [33]. However, the complexity and heterogeneity of TNBC still leave patients with an increased risk of recurrence and metastasis [34].

Tumor growth and distant metastasis are inseparable from angiogenesis. The current anti-angiogenesis targeted drugs for VEGF mainly focus on the link of inhibiting VEGF or VEGFR, such as Apatinib and macromolecular bevacizumab [35, 36]. However, studies showed that traditional anti-angiogenesis therapies are not sufficient to cut off the growth of tumor. The discovery of VM explains the failure [37]. VM is a brand-new blood supply model for highly aggressive cancer, and it could produce vascular-like structures without relying on endothelial cells. VM occurs in highly aggressive tumors, such as ovarian cancer, liver cancer, malignant melanoma, and glioma [38]. Studies confirmed that VM is closely related to tumor growth, invasion, metastasis, and prognosis [39, 40].

Breast cancer belongs to the category of “breast rock” in TCM. In recent years, TCM has achieved certain curative effects in the treatment of breast cancer. The principles of TCM are “strengthening the body” and “eliminating the evil.” “Strengthening the body” is aimed at alleviating the response and complications of surgery, chemotherapy, and radiotherapy. Recurrence and metastasis rates could be reduced by eliminating the evil. Therefore, TCM significantly improves the quality of life, increases the survival rate, and prolongs the survival time of patients.

The effects of candidate active ingredients quercetin, kaempferol, stigmasterol, and β-sitosterol in XLLXF on TNBC have been reported, which were screened by network pharmacology. Quercetin attenuates the cardiotoxicity of doxorubicin-cyclophosphamide regimen and potentiates its chemotherapeutic effect against triple-negative breast cancer [41]. Quercetin regulated the immunomodulatory function through the JAK/STAT1 signaling pathway, which was followed by the synergistic killing of breast cancer cells [42]. Quercetin could inhibit the proliferation and invasion of breast cancer cells by downregulating the expression of MMP2 and MMP9 [43]. Kaempferol suppressed proliferation and induced cell cycle arrest, apoptosis, and DNA damage in MDA-MB-231 cells [44]. Studies showed that kaempferol could effectively inhibit triclosan-induced EMT and metastatic proteins in breast cancer [45]. A notable detail that kaempferol inhibiting the invasion of MDA-MB-231 cells was related to MMPs. Kaempferol inhibited cancer cell invasion by blocking the PKCδ/MAPK/AP-1 cascade and subsequently, MMP9 expression and its activity [46]. Stigmasterol possessed significant anticancer potential, and it could be effective in the prevention and treatment of breast cancer [47]. VEGFA, PLAU, MMP2, MMP9, and MMP14 expression levels were reduced by stigmasterol treatment, which exerted a complex anticancer effect in the context of ovarian cancer [48]. β-Sitosterol induced G1 arrest and caused depolarization of mitochondrial membrane potential in breast carcinoma MDA-MB-231 cells [49]. A recent study demonstrated that encapsulation of β-sitosterol in PLGA nanoparticles is a promising strategy to exert its anticancer activity against breast cancer cells [50].

The VEGF signaling pathway is positively correlated with the formation of blood vessels and VM. VEGF can promote the proliferation and metastasis of endothelial cells by specifical effects [51]. VEGF inhibitors can effectively inhibit the growth of blood vessels by reducing the binding of VEGF and receptors [52]. A study confirmed that VEGFR1 is the VEGF receptor involved in signal transduction during the formation of VM [53]. As one of the main members of VEGF, VEGFA could also mediate VM and angiogenesis in melanoma cells [54, 55].

VEGF activated the downstream targets of PI3K pathways in the tumor microenvironment by binding to VEGFR2, including membrane type-1 (MT1-MMP) and MMP2, ultimately leading to the formation of the VM structure [56]. The various protein components in the extracellular matrix play a key role in tumor invasion and metastasis by destroying the histological barrier [57]. The expression of MMPs is high, thus promoting tumor metastasis and inducing the formation of VM ducts in microenvironments. MMP2 is an important member of MMPs, and it is located at the protruding part of the matrix. The role of MMP2 is increasingly valued, and it is considered the main proteolysis in this process enzyme [58]. MMP2 could also degrade various extracellular matrix proteins and promote the formation of VM [59].

Maniotis found that some ducts of various shapes surrounded by tumor cells were positive after iodic acid Schiff reaction staining under a light microscope in 1999 [60]. In the present study, a 3D culture model was constructed to verify if XLLXF could inhibit the formation of net-like structures in MDA-MB-231 cells in vitro. Electron microscopy and PAS-CD34 double staining experiment were conducted in vivo. Under electron microscopic observation, the red blood cells between the cells were significantly reduced after the intervention of XLLXF. Only vacuoles could be observed in the XLLXF group. The PAS-CD34 double staining experiment showed the same results. The VM structures with negative CD34 staining and positive PAS staining were significantly reduced in the XLLXF group. XLLXF could inhibit the expression of VEGFA, MMP2, MMP9, Vimentin, VE-cadherin, and Twist1 and increase that of E-cadherin, TIMP-1, and TIMP-3 in vivo and in vitro. Taken together, these results illustrated that XLLXF could inhibit the formation of VM in TNBC, and the mechanism may be related to downregulating the expression of the VEGF/MMP signal pathway.

This study has several limitations. First, the active ingredients were strictly searched and screened from databases, but XLLXF has many small compounds. HPLC only detected the top four ingredients in this study. The other main active components should be identified in the following works. Second, molecular docking technology and experimental models were applied to validate the predicted results, and the anti-TNBC mechanism of XLLXF was revealed. Due to limited funding, multiple verification may be lacking, especially the specific mechanism of the active ingredients on TNBC.

## Conclusions

In summary, the anti-TNBC mechanism of XLLXF was explored by combining network pharmacology and experimental verification. The candidate active ingredients quercetin, kaempferol, stigmasterol, and β-sitosterol were obtained through the “XLLXF–active ingredients–targets” network. Meanwhile, the potential therapeutic targets VEGFA and MMP2 were obtained through the PPI network. The key signaling pathway of VEGF/MMPs was discovered through enrichment analysis and “XLLXF–active ingredients–targets related to TNBC–key pathways” network. Then, molecular docking confirmed that quercetin, kaempferol, stigmasterol, and β-sitosterol and VEGFA and MMP2 could be stably combined. However, further studies are needed. Experimental evidence revealed that XLLXF notably reduced the formation of VM in TNBC. The therapeutic effects of XLLXF on TNBC may be related to downregulating the expression levels of VEGFA and MMP2. These findings provide direct evidence for TCM therapy in the prevention and treatment of TNBC.

## Supplementary Information

**Additional file 1**: **Table S1**. The specific informations of the active ingredients in XLLXF

**Additional file 2**: **Table S2**. The potential targets of XLLXF in the treatment of TNBC.

**Additional file 3**: **Table S3**. The top 20 active ingredients

**Additional file 4**: **Table S4**. A list of the 4 major compounds of XLLXF identifed by HPLC

**Additional file 5**: **Table S5** Target protein docking results for compounds

**Additional file 6: Fig. S1**. XLLXF inhibition of VM formation in MDA-MB-231 cells in vitro. Cells elongated and protruded pseudopodia to form net-like structures, whichwere blocked by XLLXF treatment after 24 h.

## Data Availability

The datasets used and/or analyzed during the current study are available from. the corresponding author upon reasonable request.

## Abbreviations

TNBC: Triple negative breast cancer
ER: Estrogen receptor
PR: Progesterone receptor
HER-2: human epidermal growth factor receptor-2
VM: Vasculogenic mimicry
EMT: Epithelial–mesenchymal transition
TCM: Traditional Chinese medicine
XLLXF: Xian-ling-lian-xia-fang
PPI: Protein–protein interaction
OB: Oral bioavailability
DL: Drug-likeness
DMEM: Ulbecco’s Modified Eagle Medium
MTT: 3-(4,5)- dimethylthiazolyl-3,5-diphenyltetrazolium bromide
OD: Optical density
DAPI: 4,6-diamidino-2-phenylindole
BP: Biological process
CC: Cellular component
MF: Molecular function
IHC: Immunohistochemical staining
VEGF: Vascular endothelial growth factor
VEGFA: Vascular endothelial growth factor A
VEGFR: Vascular endothelial growth factor receptor 2
MT1-MMP: Membrane type-1- matrix metalloproteinase
MMP2: Matrix metalloproteinase-2
MMP9: Matrix metalloproteinase-9
HPLC: High performance liquid chromatography
ELISA: Enzyme-linked immunosorbent assay
TIMP-1: Tissue inhibitors of metalloproteinase 1
TIMP-3: Tissue inhibitors of metalloproteinase 3
GO: Gene ontology
KEGG: Kyoto Encyclopedia of genes and genomes.
