# Supplementary material for: Investigating the mechanism of Xian-ling-lian-xia-fang for inhibiting vasculogenic mimicry in triple negative breast cancer via blocking VEGF/MMPs pathway
Source: Chin Med. 2022 Apr 4;17:44. doi: 10.1186/s13020-022-00597-5 (PMC8981688; doi:10.1186/s13020-022-00597-5)
Supplement: Supplementary file 1 — Additional file 1: Table S1. The specific informations of the active ingredients in XLLXF [file 13020_2022_597_MOESM1_ESM.pdf]

Supplementary Table 1 The specific informations of the active ingredients in XLLXF

TCM	MOL_ID	molecule_name	OB (%)	DL
**Common ingredients**	MOL000006	luteolin	36.16	0.25
	MOL000449	Stigmasterol	43.83	0.76
	MOL004355	Spinasterol	42.98	0.76
	MOL006774	stigmast-7-enol	37.42	0.75
	MOL000296	hederagenin	36.91	0.75
	MOL000098	quercetin	46.43	0.28
	MOL000359	sitosterol	36.91	0.75
	MOL000422	kaempferol	41.88	0.24
	MOL000358	beta-sitosterol	36.91	0.75
Codonopsis pilosula (Franch.) Nannf.	MOL001006	poriferasta-7,22E-dien-3beta-ol	42.98	0.76
	MOL002140	Perlolyrine	65.95	0.27
	MOL002879	Diop	43.59	0.39
	MOL003036	ZINC03978781	43.83	0.76
	MOL003896	7-Methoxy-2-methyl isoflavone	42.56	0.20
	MOL004492	Chrysanthemaxanthin	38.72	0.58
	MOL005321	Frutinone A	65.90	0.34
	MOL006554	Taraxerol	38.40	0.77
	MOL007059	3-beta-Hydroxymethyllenetanshiquinone	32.16	0.41
	MOL007514	methyl icosa-11,14-dienoate	39.67	0.23
	MOL008391	5alpha-Stigmastan-3,6-dione	33.12	0.79
	MOL008393	7-(beta-Xylosyl)cephalomannine_qt	38.33	0.29
	MOL008397	Daturilin	50.37	0.77
	MOL008400	glycitein	50.48	0.24
	MOL008406	Spinoside A	39.97	0.40
	MOL008407	(8S,9S,10R,13R,14S,17R)-17-[(E,2R,5S)-5-ethyl-6-methylhept-3-en-2-yl]-1	45.40	0.76

		0,13-dimethyl-1,2,4,7,8,9,11,12,14,15,16,17-dodecahydrocyclopenta[a]phenanthren-3-one		
	MOL008411	11-Hydroxyrankinidine	40.00	0.66
	MOL000273	(2R)-2-[(3S,5R,10S,13R,14R,16R,17R)-3,16-dihydroxy-4,4,10,13,14-pentamethyl-2,3,5,6,12,15,16,17-octahydro-1H-cyclopenta[a]phenanthren-17-yl]-6-methylhept-5-enoic acid	30.93	0.81
	MOL000275	trametenolic acid	38.71	0.80
	MOL000276	7,9(11)-dehydropachymic acid	35.11	0.81
	MOL000279	Cerevisterol	37.96	0.77
	MOL000280	(2R)-2-[(3S,5R,10S,13R,14R,16R,17R)-3,16-dihydroxy-4,4,10,13,14-pentamethyl-2,3,5,6,12,15,16,17-octahydro-1H-cyclopenta[a]phenanthren-17-yl]-5-isopropyl-hex-5-enoic acid	31.07	0.82
Poria cocos(Schw.) Wolf	MOL000282	ergosta-7,22E-dien-3beta-ol	43.51	0.72
	MOL000283	Ergosterol peroxide	40.36	0.81
	MOL000285	(2R)-2-[(5R,10S,13R,14R,16R,17R)-16-hydroxy-3-keto-4,4,10,13,14-pentamethyl-1,2,5,6,12,15,16,17-octahydrocyclopenta[a]phenanthren-17-yl]-5-isopropyl-hex-5-enoic acid	38.26	0.82
	MOL000287	3beta-Hydroxy-24-methylene-8-lanostene-21-oic acid	38.70	0.81
	MOL000289	pachymic acid	33.63	0.81
	MOL000290	Poricoic acid A	30.61	0.76
	MOL000291	Poricoic acid B	30.52	0.75

	MOL000292	poricoic acid C	38.15	0.75
	MOL000300	dehydroeburicoic acid	44.17	0.83
Epimedium brevicornu Maxim.	MOL000622	Magnograndiolide	63.71	0.19
	MOL001510	24-epicampesterol	37.58	0.71
	MOL001645	Linoleyl acetate	42.10	0.20
	MOL001771	poriferast-5-en-3beta-ol	36.91	0.75
	MOL001792	DFV	32.76	0.18
	MOL003044	Chryseriol	35.85	0.27
	MOL003542	8-Isopentenyl-kaempferol	38.04	0.39
	MOL004367	olivil	62.23	0.41
	MOL004373	Anhydroicaritin	45.41	0.44
	MOL004380	C-Homoerythrinan, 1,6-didehydro-3,15,16-trimethoxy-, (3.beta.)-	39.14	0.49
	MOL004382	Yinyanghuo A	56.96	0.77
	MOL004384	Yinyanghuo C	45.67	0.50
	MOL004386	Yinyanghuo E	51.63	0.55
	MOL004388	6-hydroxy-11,12-dimethoxy-2,2-dimethyl-1,8-dioxo-2,3,4,8-tetrahydro-1H-isochromeno[3,4-h]isoquinolin-2-ium	60.64	0.66
	MOL004391	8-(3-methylbut-2-enyl)-2-phenyl-chromone	48.54	0.25
	MOL004394	Anhydroicaritin-3-O-alpha-L-rhamnoside	41.58	0.61
	MOL004396	1,2-bis(4-hydroxy-3-methoxyphenyl)propan-1,3-diol	52.31	0.22
	MOL004425	Icariin	41.58	0.61
	MOL004427	Icariside A7	31.91	0.86
	MOL000902	Curcumol	103.55	0.13

	MOL000889	Isocurcumenol	97.67	0.13
	MOL000901	Curcumenol	87.82	0.13
	MOL000940	Bisdemethoxycurcumin	77.38	0.26
	MOL004245	Curcumadiol	60.27	0.10
	MOL000900	(5R,6R)-5-isopropenyl-3,6-dimethyl-6-vinyl-5,7-dihydrobenzofuran-4-one	57.05	0.11
	MOL000208	(+)-Aromadendrene	55.74	0.10
	MOL000935	Hepanal	53.83	0.10
	MOL000938	Calarene	51.55	0.11
	MOL000937	58870_FLUKA	49.01	0.10
	MOL000906	Wenjine	47.93	0.27
Curcuma phaeocaulis Valeton	MOL000903	Gweicurculactone	42.92	0.14
	MOL004255	Curdione	38.94	0.08
	MOL000897	(3S,3aR,8aR)-3,8a-dihydroxy-5-isopropylidene-3,8-dimethyl-1,2,3a,4-tetrahydroazulen-6-one	38.70	0.12
	MOL000891	(1R,10R)-epoxy-1,10-dihydrocurdione	36.73	0.12
	MOL000474	(-)-Epoxycaryophyllene	35.94	0.13
	MOL000898	Curcumenone	34.17	0.11
	MOL000910	Germacrone	32.50	0.07
	MOL000915	(1S,10S),(4S,5S)-germacrone-1(10),4-diepoxide	30.48	0.18
	MOL000908	Beta-elemene	25.63	0.06
	MOL003770	Daucosterol	20.46	0.65
	MOL000090	Curcumin	5.15	0.41
	MOL001603	Demethoxycurcumin	4.37	0.33
	MOL000173	wogonin	30.68	0.23
	MOL000351	Rhamnazin	47.14	0.34

	MOL000953	CLR	37.87	0.68
	MOL001040	(2R)-5,7-dihydroxy-2-(4-hydroxyphenyl)chroman-4-one	42.36	0.21
	MOL001735	Dinatin	30.97	0.27
	MOL001755	24-Ethylcholest-4-en-3-one	36.08	0.76
	MOL001973	Sitosteryl acetate	40.39	0.85
	MOL002714	baicalein	33.52	0.21
Scutellaria barbata D. Don	MOL002719	6-Hydroxynaringenin	33.23	0.24
	MOL002776	Baicalin	40.12	0.75
	MOL002915	Salvigenin	49.07	0.33
	MOL005190	eriodictyol	71.79	0.24
	MOL005869	daucostero_qt	36.91	0.75
	MOL008206	Moslosooflavone	44.09	0.25
	MOL012245	5,7,4'-trihydroxy-6-methoxyflavanone	36.63	0.27
	MOL012246	5,7,4'-trihydroxy-8-methoxyflavanone	74.24	0.26
	MOL012248	5-hydroxy-7,8-dimethoxy-2-(4-methoxyphenyl)chromone	65.82	0.33
	MOL012250	7-hydroxy-5,8-dimethoxy-2-phenyl-chromone	43.72	0.25
	MOL012251	Chrysin-5-methylether	37.27	0.20
	MOL012252	9,19-cyclolanost-24-en-3-ol	38.69	0.78
	MOL012254	campesterol	37.58	0.71
	MOL012266	rivularin	37.94	0.37
	MOL012269	Stigmasta-5,22-dien-3-ol-acetate	46.44	0.86
	MOL012270	Stigmastan-3,5,22-triene	45.03	0.71
	MOL000737	morin	46.23	0.27
Prunella vulgaris L	MOL004798	delphinidin	40.63	0.28
	MOL006767	Vulgaxanthin-I	56.14	0.26
	MOL006772	poriferasterol monoglucoside_qt	43.83	0.76
