# Supplementary material for: Investigating the mechanism of Xian-ling-lian-xia-fang for inhibiting vasculogenic mimicry in triple negative breast cancer via blocking VEGF/MMPs pathway
Source: Chin Med. 2022 Apr 4;17:44. doi: 10.1186/s13020-022-00597-5 (PMC8981688; doi:10.1186/s13020-022-00597-5)
Supplement: Supplementary file 2 — Additional file 2: Table S2. The potential targets of XLLXF in the treatment of TNBC. [file 13020_2022_597_MOESM2_ESM.pdf]

Supplementary Table 2 The potential targets of XLLXF in the treatment of TNBC

| No. | gene | No. | gene | No. | gene |
|---|---|---|---|---|---|
| 1 | TP53 | 20 | GJA1 | 39 | CCNA2 |
| 2 | ESR1 | 21 | AHR | 40 | NQO1 |
| 3 | EGFR | 22 | NOS2 | 41 | ABCC1 |
| 4 | VEGFA | 23 | INSR | 42 | CYCS |
| 5 | TNF | 24 | ODC1 | 43 | PIK3CG |
| 6 | IL6 | 25 | MAPK1 | 44 | MPO |
| 7 | AR | 26 | JUN | 45 | TOP1 |
| 8 | RB1 | 27 | IL2 | 46 | MMP3 |
| 9 | EGF | 28 | PTGS2 | 47 | NR3C1 |
| 10 | MET | 29 | MMP2 | 48 | CASP7 |
| 11 | IL1B | 30 | CCL2 | 49 | MMP13 |
| 12 | ESR2 | 31 | NR3C2 | 50 | NCOA1 |
| 13 | PLAU | 32 | GSTP1 | 51 | NCOA2 |
| 14 | PPARG | 33 | MAPK8 | 52 | AKR1C3 |
| 15 | MMP1 | 34 | KDR | 53 | CA2 |
| 16 | PGR | 35 | CTSD | 54 | COL1A1 |
| 17 | CHEK1 | 36 | GSK3B | 55 | SOD1 |
| 18 | BCL2 | 37 | GSTM1 | | |
| 19 | IFNG | 38 | MAPK14 | | |
