# Supplementary material for: Investigating the mechanism of Xian-ling-lian-xia-fang for inhibiting vasculogenic mimicry in triple negative breast cancer via blocking VEGF/MMPs pathway
Source: Chin Med. 2022 Apr 4;17:44. doi: 10.1186/s13020-022-00597-5 (PMC8981688; doi:10.1186/s13020-022-00597-5)
Supplement: Supplementary file 3 — Additional file 3: Table S3. The top 20 active ingredients [file 13020_2022_597_MOESM3_ESM.pdf]

Supplementary table 3 The top 20 active ingredients

MOL_ID	Compounds	Degree	Belongs
MOL000098	quercetin	135	*Epimedium brevicornu Maxim., Scutellaria barbata D. Don, Prunella vulgaris L*
MOL000422	kaempferol	52	*Epimedium brevicornu Maxim., Prunella vulgaris L*
MOL000449	Stigmasterol	42	*Codonopsis pilosula Nannf., Scutellaria barbata D. Don, Prunella vulgaris L*
MOL000358	beta-sitosterol	38	*Scutellaria barbata D. Don, Prunella vulgaris L*
MOL000173	wogonin	21	*Scutellaria barbata D. Don*
MOL002714	baicalein	21	*Scutellaria barbata D. Don*
MOL000296	hederagenin	19	*Poria cocos Wolf, Curcumae Rhizoma*
MOL004355	Spinasterol	16	*Codonopsis pilosula Nannf., Prunella vulgaris L*
MOL000359	sitosterol	16	*Epimedium brevicornu Maxim., Scutellaria barbata D. Don*
MOL004373	Anhydroicaritin	15	*Epimedium brevicornu Maxim.*
MOL001735	Dinatin	15	*Scutellaria barbata D. Don*
MOL012266	rivularin	15	*Scutellaria barbata D. Don*
MOL003896	7-Methoxy-2-methyl isoflavone	14	*Codonopsis pilosula Nannf.*
MOL008400	glycitein	14	*Codonopsis pilosula Nannf.*
MOL003542	8-Isopentenyl-kaempferol	14	*Epimedium brevicornu Maxim.*
MOL000351	Rhamnazin	14	*Scutellaria barbata D. Don*
MOL001040	(2R)-5,7-dihydroxy-2-(4-hydroxyphenyl)chroman-4-one	14	*Scutellaria barbata D. Don*
MOL005190	eriodictyol	14	*Scutellaria barbata D. Don*

MOL008206	Moslosooflavone	14	*Scutellaria barbata D. Don*
MOL012248	5-hydroxy-7,8-dimethoxy-2-(4-methoxyphenyl)chromone	14	*Scutellaria barbata D. Don*
