# Supplementary material for: Investigating the mechanism of Xian-ling-lian-xia-fang for inhibiting vasculogenic mimicry in triple negative breast cancer via blocking VEGF/MMPs pathway
Source: Chin Med. 2022 Apr 4;17:44. doi: 10.1186/s13020-022-00597-5 (PMC8981688; doi:10.1186/s13020-022-00597-5)
Supplement: Supplementary file 4 — Additional file 4: Table S4. A list of the 4 major compounds of XLLXF identifed by HPLC [file 13020_2022_597_MOESM4_ESM.pdf]

Supplementary table 4  A list of the 4 major compounds of XLLXF identifed by HPLC

| No. | Name | Time | Area | Structure |
|---|---|---|---|---|
| 1 | Quercetin | 7.73 | 12558 | 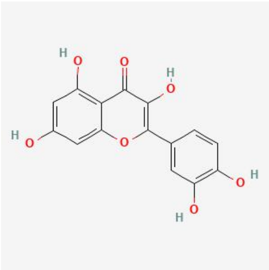 |
| 2 | Kaempferol | 8.24 | 9738 | 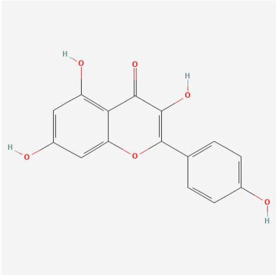 |
| 3 | Stigmasterol | 10.05 | 15545 | 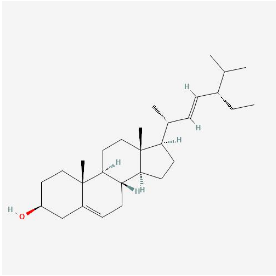 |
| 4 | β-sitosterol | 14.15 | 26489 | 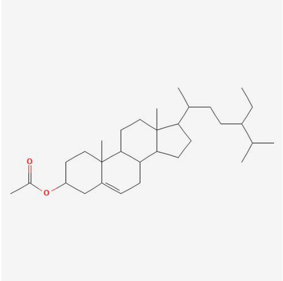 |
