# Supplementary material for: Investigating the mechanism of Xian-ling-lian-xia-fang for inhibiting vasculogenic mimicry in triple negative breast cancer via blocking VEGF/MMPs pathway
Source: Chin Med. 2022 Apr 4;17:44. doi: 10.1186/s13020-022-00597-5 (PMC8981688; doi:10.1186/s13020-022-00597-5)
Supplement: Supplementary file 5 — Additional file 5: Table S5 Target protein docking results for compounds [file 13020_2022_597_MOESM5_ESM.pdf]

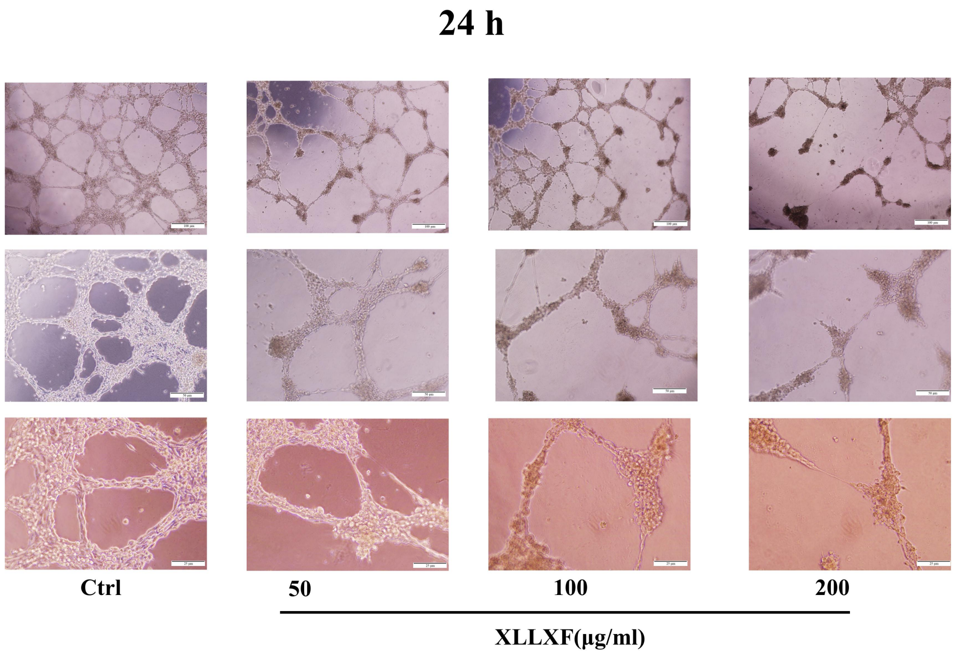

Supplementary Fig.1 XLLXF inhibition of VM formation in MDA-MB-231 cells in *vitro*. Cells elongated and protruded pseudopodia to form net-like structures, which were blocked by XLLXF treatment after 24 h.
