# Supplementary material for: Investigating the mechanism of Xian-ling-lian-xia-fang for inhibiting vasculogenic mimicry in triple negative breast cancer via blocking VEGF/MMPs pathway
Source: Chin Med. 2022 Apr 4;17:44. doi: 10.1186/s13020-022-00597-5 (PMC8981688; doi:10.1186/s13020-022-00597-5)
Supplement: Supplementary file 6 — Additional file 6: Fig. S1. XLLXF inhibition of VM formation in MDA-MB-231 cells in vitro. Cells elongated and protruded pseudopodia to form net-like structures, whichwere blocked by XLLXF treatment after 24 h. [file 13020_2022_597_MOESM6_ESM.pdf]

Supplementary table 5 Target protein docking results for compounds

Compound structure	Name	Binding energy (kcal/mol)	Combination type
Quercetin	MMP2	-8.17	Hydrogen bonds, Hydrophobic interactive
	VEGFA	-7.59	Hydrogen bonds, Hydrophobic interactive
Kaempferol	MMP2	-7.81	Hydrogen bonds, Hydrophobic interactive
	VEGFA	-7.32	Hydrogen bonds, Hydrophobic interactive
beta-Sitosterol	MMP2	-6.93	Hydrogen bonds, Hydrophobic interactive
	VEGFA	6.48	Hydrogen bonds, Hydrophobic interactive
Stigmasterol	MMP2	-6.67	Hydrogen bonds, Hydrophobic interactive
	VEGFA	-6.72	Hydrogen bonds, Hydrophobic interactive
